# MODs vs. NPs: Vying for the Future of Printed Electronics

**DOI:** 10.1002/chem.202004860

**Published:** 2021-03-01

**Authors:** Samuel P. Douglas, Shreya Mrig, Caroline E. Knapp

**Affiliations:** ^1^ Department of Chemistry University College London 20 Gordon Street London WC1H 0AJ UK

**Keywords:** conductivity, inks, nanoparticle, precursor, printing

## Abstract

This Minireview compares two distinct ink types, namely metal‐organic decomposition (MOD) and nanoparticle (NP) formulations, for use in the printing of some of the most conductive elements: silver, copper and aluminium. Printing of highly conductive features has found purpose across a broad array of electronics and as processing times and temperatures reduce, the avenues of application expand to low‐cost flexible substrates, materials for wearable devices and beyond. Printing techniques such as screen, aerosol jet and inkjet printing are scalable, solution‐based processes that historically have employed NP formulations to achieve low resistivity coatings printed at high resolution. Since the turn of the century, the rise in MOD inks has vastly extended the range of potentially applicable compounds that can be printed, whilst simultaneously addressing shelf life and sintering issues. A brief introduction to the field and requirements of an ink will be presented followed by a detailed discussion of a wide array of synthetic routes to both MOD and NP inks. Unindustrialized materials will be discussed, with the challenges and outlook considered for the market leaders: silver and copper, in comparison with the emerging field of aluminium inks.

## Graphical abstract

**Fresh ink**: This minireview considers the highlights and challenges facing both the precursor (MOD) and nanoparticle (NP) approach toward the printing of highly conductive metallic features. Silver, copper and aluminium are some of the most conductive elements and their printing has been applied to a broad field of electronics. Up‐to‐date synthetic routes to MOD and NP formulations are presented and compared to the fundamental requirements of an ink.

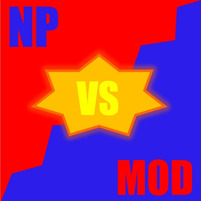

## 1 Introduction

The sector for printed electronics has seen huge growth in the last few decades. Devices such as thin‐film transistors,^[1]^ radio‐frequency identification devices,^[2]^ light emitting diodes (LED) displays,^[3]^ sensors,^[4]^ batteries,^[5]^ solar cells,^[6]^ have become paramount in society and as such, industry leaders are looking for innovative approaches to make their processes more material‐, time‐ and cost‐efficient. One such method of achieving these targets is to deposit the conductive circuits found in these devices using metal containing inks.

Traditionally, metallic features of conventional electronics were deposited using methods like chemical vapour deposition (CVD),^[7]^ sputtering^[8]^ and laser beam evaporation.^[9]^ However, these techniques often involve numerous steps, generate large amounts of toxic waste and are costly. Additionally, the high processing temperatures and/or the necessity for reduced pressure in these techniques has limited the use of increasingly popular low‐cost flexible substrates such as plastics, paper, and textiles.^[10]^

Deposition of metal circuits via the solution‐based processing of metal inks is more common due to the relative ease, moderate conditions, and adaptability of the techniques. Printing is a popular choice for solution‐based metal deposition, although drop, spin, dip, and spray coating, are also commonplace in the literature. Printing processes, specifically inkjet printing and additive manufacturing (3D printing), have undergone a technological surge since the turn of the century, with no other manufacturing technologies offering such versatility.^[11]^ For most researchers in this field, depositing highly conductive metallic films via inkjet printing is their ultimate objective, as along with this technique's versatility, it is a highly efficient process.

Metal inks can be categorized into two main types: metal‐organic precursor formulations, known as metal‐organic decomposition (MOD) inks and nanoparticle (NP) formulations. Building on the review published by Kamyshny, Steinke and Magdassi in early 2011,^[12]^ this article presents an up‐to‐date report of both MOD and NP inks containing some of the most conductive metallic elements; silver (Ag), copper (Cu) and aluminium (Al), concluding with a future outlook for this field.

## 2 Overview of Requirements

Metal inks are generally comprised of a dispersed or dissolved metal‐based component, in a liquid vehicle (solvent) that typically determines the basic properties of the ink such as viscosity, surface tension and wettability. It is also not unusual for inks to include a resin binder to improve film adhesion.

The most essential requirement of a metal ink is that once deposited and processed, coatings should display good electrical conductivity (or low resistivity (*ρ*))—this is why the most conductive elements are used. Ag has the lowest (bulk) resistivity of all elements with a value of 1.59×10^−8^ Ω m, Cu is second (1.72×10^−8^ Ω m), third is gold (2.44×10^−8^ Ω m) followed by Al (2.65×10^−8^ Ω m).^[13]^ Regarding price, as of 2021 gold's cost is approximately 75 times that of Ag, 7500 times Cu and 30,000 times that of Al; limiting gold's industrial use.

Ideally, processing techniques such as annealing, curing and sintering should be undertaken at low temperatures (<150 °C), as to enable the deposition of material onto low‐cost flexible substrates such as plastics, textiles, and paper. For printing, metal inks should be stable enough to retain a good shelf life, have properties than enable good printability and film resolution as well as, be highly resistant to aggregation and precipitation, such that minimal printer maintenance is required. Cost and environmental impact are also important considerations for industrial scale up, so inks should be loaded with as high a weight (wt.)% of the metal as possible, to minimize wastage. High resolution of printed features is desirable, and coatings need to be strongly adhered to the substrate being able to withstand bending. Finally, this should all ideally take place at high speed, such that it is compatible with existing printing technology, e.g., the industrial roll‐to‐roll process. In summary:

‐Low  resistivity: deposited metal coatings should have a resistivity comparable to the bulk metal

‐Low  temperature: inks should transform to the metal and sinter at temperatures compatible with flexible substrates (<150 °C)

‐Long  shelf life of inks

‐Low  cost and environmental impact

‐High  wt.% of metal in inks

‐Short  sintering times

‐High  resolution of printed features

‐Good  adhesion of metal coatings to allow for bending

Shelf life, or good stability is likely to be inversely proportional to low temperature, high speed conversion; indicating that a compromise may need to be made when considering the ideal specifications listed above. At this point there are two avenues to consider when designing the perfect ink, either a discreet molecule in a MOD ink, or a NP system. There have been several developments in recent years and this Minireview will highlight some of the most important discoveries using non‐commercial MOD and NP inks; all details are summarized in Tables S1—5 in Supporting Information.

### 2.1 Metal‐organic decomposition (MOD) inks

**Scheme 1:**
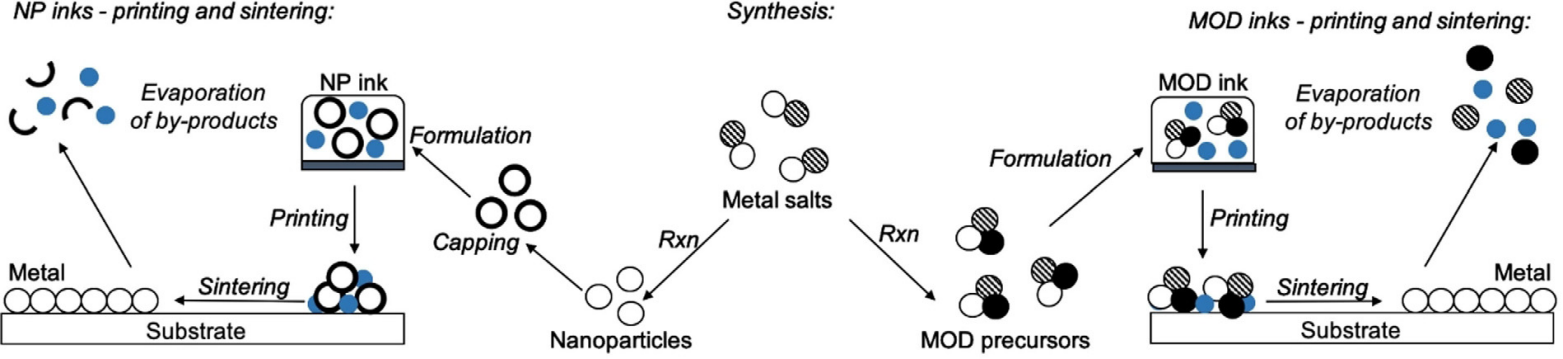
MODs vs. NP: From the centre, schematic illustration showing the different synthetic routes to either NP (to the left) or MOD precursors (to the right), bookended by the process involved in printing and sintering metal coatings.

MOD inks contain metal‐organic precursors, usually dissolved in solvent. They can be synthesised from commercially available metal salts and their decomposition profile tailored by choice of coordinating ligand. Colloidal stabilising agents are not required, meaning that these inks often can be sintered at lower temperatures, however inks of this type are usually associated with lower metal wt.% loading due to additional coordinating groups. Solvents in these inks can act as spectators or as complexing agents, for example, amines,^[14]^ alkanolamines^[15]^ or carboxylic acids.^[16]^ These compounds contain metal centres in positive oxidation states, so upon decomposition there is a necessity for the centre to undergo reduction (most likely via oxidation of the salt anion and/or coordinating groups) before nucleation and growth of ‘particulates’ can occur (Scheme 1). It is not uncommon in the literature for these sintered particulates to be referred to as ‘particles’, particularly when describing scanning electron microscopy (SEM) images, for example. In the interest of clarity, since nanoparticles are discussed at length in this Minireview, the term ‘particulates’ will be exclusively used to refer to the nucleation and growth of converted MOD inks to coatings.

### 2.2 Nanoparticle (NP) inks

Currently, most commercial metal inks are NP based. NP inks can be made from the reduction of commercially available metal salts, followed by a stabilising step, such as capping (Scheme 1). Inks consist of metal NPs dispersed in solvent with the use of additional colloidal stabilising agents owing to the high likelihood of NP aggregation and/or precipitation. NP inks are normally associated with higher wt.% loading than their MOD counterparts, however aggregation and/or precipitation of NPs can lead to drastic decreases in weight loading and nozzle clogging. Stabilising agents, which are often polymeric in nature, require higher temperatures to remove.

## 3 Recent Developments in Metal Inks

### 3.1 Silver inks

Presently, Ag is the metal that has been most utilized in conductive inks due to its excellent conductivity (*ρ*=1.59×10^−8^ Ω m), moderate price and relatively strong resistance to oxidation due to its high standard electrode potential. It has been widely reported in both the MOD and NP literature.

#### 3.1.1 Silver MOD inks

Although not as commonplace as their NP analogues, Ag MOD formulations still occupy a presence in the metallic conductive inks literature and have been known since the early 1980s.^[17]^ Initially, most organo‐silver complexes synthesised for metal deposition were centred on silver‐oxygen bonds, including carboxylates and diketonates, however compounds with silver‐nitrogen bonds have become increasingly common. Typically, non‐commercial Ag compounds are synthesised from silver nitrate (AgNO_3_) as a starting material. Aqueous AgNO_3_ inks themselves are light‐sensitive and have high decomposition temperatures (≈440 °C) and hence are not particularly useful.

**Figure 1:**
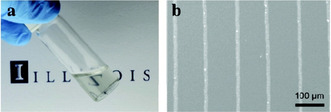
(a) Image of the reactive Ag ink. (b) Conductive Ag lines printed on a silicon substrate via direct‐write assembly using a 100 nm nozzle. Reproduced with permission from ref. [16], Copyright 2012, American Chemical Society.

There have been a number of interesting developments over the last decade, with many derived from the work of Walker et al. in 2012,^[16]^ who produced a reactive Ag ink via a modified Tollens’ process, using silver acetate, ammonium hydroxide and formic acid. The reactive Ag ink contained 22 wt.% Ag, was highly transparent and printed using ultrafine nozzles (Figure 1). The ink's high reactivity allowed it to form Ag metal at room temperature (RT) after approx. 1 day, although a high resistivity metallic film was produced, due to the presence of undecomposed silver acetate. More promisingly, the ink was sintered at 90 °C for 15 min, producing a film with an electrical resistivity close to that of bulk Ag (*ρ*=1.60×10^−8^ Ω m). It adhered well to several polymeric substrates and reasonably well to other substrates, such as glass and cellulose‐based materials. Later, Liu laser direct patterned the same ink to produce Ag films on polyimide (PI),^[18]^ which also displayed low resistivities of 2.10×10^−8^ Ω m. Later in 2016, Stempien et al. also studied Walker et al.’s ink in relation to deposition onto various textiles achieving low sheet resistance (R_s_) in the range of 0.155–0.389 Ω sq^−1^ after 8 printing cycles,^[19]^ whilst Iannaccone roll‐to‐roll printed the same ink during the manufacture of solar cells.^[20]^ Knapp et al. used the ink to coat paper which was then subject to RT, atmospheric pressure plasma sintering, which yielded highly conductive Ag films with *ρ*=2.60×10^−8^ Ω m.^[21]^ No saturation of the paper substrate was seen despite its porous nature, indicating the plasma‐assisted decomposition of the MOD ink occurs rapidly. Wang et al. most recently used Walker et al.’s versatile ink to deposit highly conductive films on PI using a combination of thermal and plasma sintering,^[22]^ producing a film with lowest *ρ=*6.00×10^−8^ Ω m, when a film annealed at 90 °C for 15 min, was exposed to 80 W plasma for 4 min. The work using Walker et al.’s ink since its fabrication aptly displays the importance of attempting different deposition and sintering techniques on the same formulation, as not all inks are as adaptable.

Around the same time as the work by Walker et al., Chen et al. formulated several ink formulations containing different ratios of aqueous solutions of silver ammonia solution and diethanolamine.^[23]^ This work presented one of the first uses of alkanolamines in Ag MOD inks, which have since become commonplace as mild Ag reducing agents at modest temperatures. Storage issues with inks containing alkanolamines were highlighted—the diethanolamine decomposed slowly generating Ag NPs gradually, with the ink turbidity slightly increasing after 1 day. After 3 days, the Ag ink became cloudy, with noticeable NP aggregation at the bottom of the vessel. Storing this ink at lower temperatures allowed it to have an increased shelf life. This is a common technique employed to improve storage life of all inks. The authors also state a less trivial silver ammonium ion synthesis—a simplified approach starting from silver oxide (Ag_2_O) and ammonia. This was done as an alternative to using AgNO_3_ as the starting reagent, as the use of AgNO_3_ can lead to nitrate salts being embedded in the thin films after ink drying which increases the resistivity of the coatings produced. Ink reactivity was fully investigated via UV absorption analysis of the 3 wt.% Ag ink stored under various temperatures and times. They state the mechanism of Ag film derives from diethanolamine degradation in the ink at temperatures >50 °C, generating formaldehyde, which reduces silver ammonia ions to form Ag thin films. Films were inkjet printed under numerous conditions, with a best *ρ*=6.00×10^−8^ Ω m (under four times that of bulk Ag) when films were heated at 75 °C for 20 min, with films displaying excellent adhesion to polyethylene terephthalate (PET) substrate.

**Figure 2:**
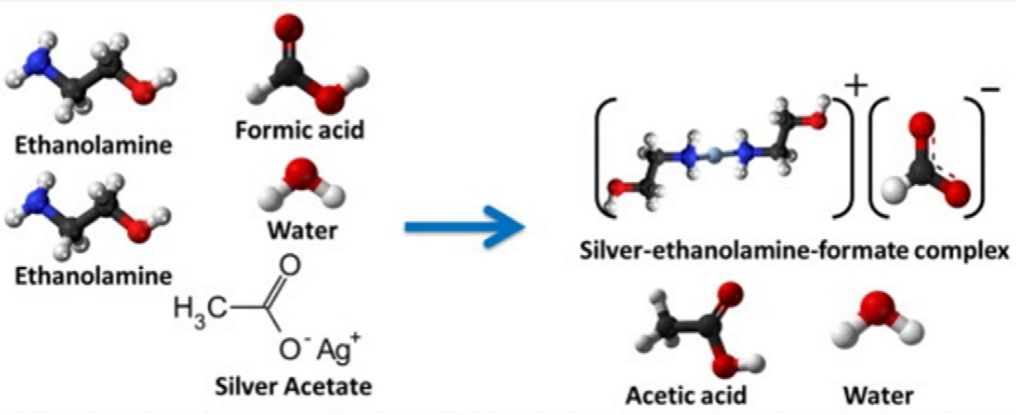
Chemical reaction involved in the formulation of Vaseem et al.’s ink. Adapted with permission from ref. [24], Copyright 2016, Elsevier.

Vaseem et al. were critical of the ink produced by Walker et al., stating that the high reactivity of the ink would be detrimental to long‐term use,^[24]^ stating that the ready decomposition of the ink at RT is actually a drawback, as this may lead to the formation of clogging Ag particles in the nozzle if inkjet printed. This work, in line with Chen et al.’s above,^[23]^ suggested that the fast vaporisation of ammonia and carbon dioxide upon sintering, leads to bubbling which may severely reduce the quality of deposits. With the aim of producing a Ag ink that was proven to be robust over longer durations of time, whilst producing high quality films, they reported a silver‐ethylamine‐ethanolamine‐formate‐complex ink, where ethylamine, ethanolamine and formate species act as in situ complexing solvents and as reducing agents (Figure 2). The 17 wt.% Ag loaded ink was transparent and stable over long durations of time. Stability was monitored by measuring the average drop mass over a period of 5 months and remained consistent. It was stable in a sealed glass vial at RT, although storage life could be increased at lower temperatures. The formulated ink was inkjet printed on numerous substrates, with films having uniform surface morphology and excellent adhesion. The ink was sintered at a temperature of 150 °C for 30 min for resistivity measurements, as no further weight loss was observed above this temperature. The conductivity of the films increased as the number of layers of deposited metal ink increased. The films produced on glass and polyethylene naphthalate (PEN) substrates had *ρ*=4.10×10^−8^ Ω m and *ρ*=4.70×10^−8^ Ω m, respectively, between 2—3 times that of bulk Ag. In 2018 Vaseem et al. followed up research on their silver‐ethylamine‐ethanolamine‐formate‐complex ink by depositing it at 80 °C onto a PI substrate via inkjet printing.^[25]^ After 8 printing layers, the metallic film had a *ρ*=5.95×10^−8^ Ω m, showing a marked improvement against the commercial Ag NP ink they also analysed. Films were subjected to bending and crushing tests, with little conductance loss after testing, verifying that the ink showed excellent tolerance to stress. However, it must be noted, in an earlier report Vaseem et al. were critical of their first ink formulation,^[26]^ stating that multicomponent solvent systems had several drawbacks such as limiting Ag wt.% loading, as well as, negatively affecting conductive film formation and ink stability. A simpler silver‐ethanolamine‐formate complex ink was deposited on numerous substrates showing a lowest *ρ*=7.14×10^−8^ Ω m on glass, when subjected to microwave plasma radiation for 1.5 min representing a considerable reduction in time. Films were also deposited using more conventional thermal sintering at 180 °C for 30 min, producing more modest results.

Bhat et al. reported a simple synthetic procedure for the development of a durable, particle‐free Ag MOD ink,^[27]^ in which ethylamine and ammonia act as the complexing agents, similar to Chen's ink, which could be sintered at low temperatures (<90 °C). Typically, silver acetate was mixed with ethylamine and ammonium hydroxide before formic acid was added producing a grey solution. Hydroxyethyl cellulose (HEC, 2 % in water‐methanol solution) was added, before filtering to produce the colourless ink, with a small amount of ethanolamine also added to prevent the nozzle tip from clogging. The ink was printed onto variable substrates including glass, PET and PI using spin‐coating and inkjet printing methods, giving smooth highly conductive Ag deposits that adhered excellently. The spin‐coated film on glass annealed at 60 °C for 1 day and the inkjet printed film on PET sintered at 75 °C for 1 h yielded low resistivities of *ρ*=9.32×10^−7^ Ω m and *ρ*=3.65×10^−7^ Ω m, respectively. Ag films were finally connected with LEDs as a proof of concept which exhibited outstanding adhesion across bending and torsional testing.

More recently in 2019, Mou et al. reported a conductive ink with good chemical stability that employed a silver‐isopropanolamine complex, formic acid reductant, and HEC adhesive agent.^[28]^ Mask printing of the ink on PI, followed by thermal sintering, produced flexible electrodes. The effects of differing sintering parameters and the content of HEC adhesive agent on the physical and microstructural properties of the deposited Ag films were systematically investigated. Ink deposited at 110 °C yielded films with *ρ=*1.21×10^−7^ Ω m, eight times higher that of bulk Ag. The sintered Ag layer also exhibited excellent flexibility and low relative resistances after the bending, twisting, and folding tests.

Following this work, Kell et al. presented a simple formulation comprising of silver neodecanoate,^[29]^ a longer chained carboxylate alternative to formate and acetate, in addition to, ethyl cellulose, and solvent, which they said was more economical and had improved performance versus established inks. The addition of ethyl cellulose to the formulation increased the viscosity of the ink. This modified rheology, allowed deposition via screen printing. Additionally, by acting as both binder and adhesive it improved the mechanical properties of the sintered traces. Thin, screen printed films with good electrical and mechanical properties were deposited on PI, dried at elevated temperatures, followed by either thermal (230 °C for 10 min) or photonic sintering, with the latter never having been previously demonstrated for silver‐salt‐based inks. Metallic films exhibited low surface roughness for thermally sintered films, as well as, submicron thickness and narrow line widths, outperforming benchmarks set by NP based inks of the time. Films were also resistant to flexing and creasing on (<10 % reduction change in resistance) due to the addition of the ethyl cellulose. The ink formulation was also applied to aerosol jet printing with a best conducting film produced on glass with *ρ*=2.80×10^−8^ Ω m. Shen et al. had previously described the use of silver neodecanoate/cellulose blends to direct‐write highly conductive Ag films on glass at sub 200 °C temperatures, with *ρ*=9.00×10^−8^ Ω m after sintering at 115 °C for 1 h, however investigations into the use of flexible substrates were not undertaken.^[30]^

Xie and co‐workers decided to investigate the effects of complexing of three different alkanolamines with an increasing number of OH groups (ethanolamine, diethanolamine and triethanolamine) to Ag.^[31]^ Three inks were synthesised using silver acetate, oleic acid and *n*‐butanol, in addition to, the complexing agent, with differing amounts of solvent, mostly likely to achieve comparable viscosities between inks. Each synthesis yielded a clear ink that was highly stable to precipitation. Inks were spin‐coated onto glass substrates, before undergoing a 10 min 100 °C low‐temperature processing, followed by a 30 min 180 °C high‐temperature processing, producing highly conductive Ag films. Their two‐step baking process was designed to produce large Ag particulates (that have increased conductivity) during the low‐temperature processing and to remove insulating organic materials between the NPs, in addition to, sintering them together during the high‐temperature processing. They state that the use of higher boiling point (BP) alkanolamines results in more uniform Ag crystalline domains forming across the Ag films, due to slower evaporation during thermal annealing, leading to more conductive films. Thus, the more conventional ethanolamine reductant was surprisingly less preferred. The authors state that diethanolamine was more desirable than triethanolamine, as the removal was more facile, so further tests concentrated on using the former as the alkanolamine of choice. Using diethanolamine as the mild reducing agent, spin‐coating a double layer of Ag, and their two‐step thermal processing technique they were able to produce a highly conductive film with *ρ*=6.67×10^−8^ Ω m, just over four times that of bulk Ag.

**Figure 3:**
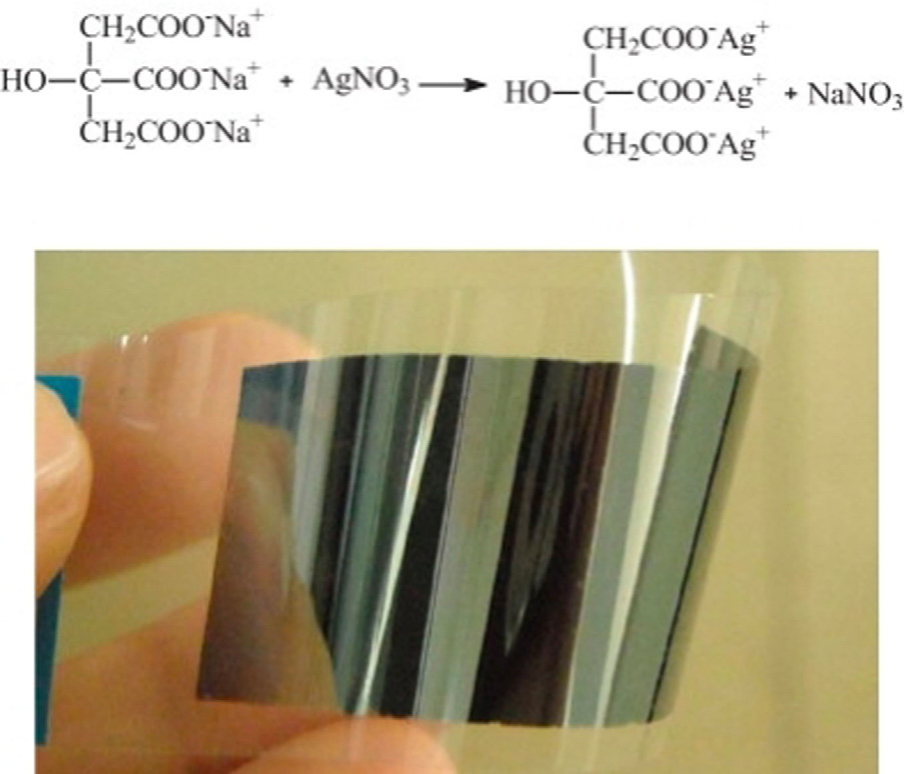
Preparation of silver citrate conductive ink, above, and photograph of inkjet printed patterns on PET substrate. Adapted with permission from ref. [32], Copyright 2012, Elsevier.

Nie et al. opted to use silver citrate as the functional material for their inks, with citrate being a branched carboxylate that bonds to three Ag cations,^[32]^ also using 1,2‐diaminopropane as the complexing agent, along with methanol and isopropanol as the media to adjust viscosity and surface tension (Figure 3). Films were inkjet printed and thermally sintered with notable results including a lowest *ρ*=1.70×10^−7^ Ω m after curing at 150 °C for 50 min and *ρ=*3.10×10^−8^ Ω m at 230 °C. The conductive films obtained exhibited exemplary adhesive properties and high reflection.

Yang et al. in 2017, also opted to use silver citrate as their metal precursor,^[33]^ stating that compared to other commonly used Ag salts, it looked to be the ideal for use in MOD inks. Silver citrate was dispersed in 2‐propanol, ethylene glycol and ethylenediamine, having an Ag content of around 10 wt.%. The ink was then drop‐casted onto PI and sintered at various temperatures between 125–200 °C (chosen due to differential scanning calorimetry (DSC) and UV/Vis analysis of the ink), for up to 1 h. Unsurprisingly, the films sintered at the highest temperature and the longest time had the lowest resistivity (*ρ*=3.90×10^−7^ Ω m at 200 °C for 1 h), with a comprehensive study conducted for both variables, including analysis of Ag nanocrystal size. Yang, having previously been critical of using silver oxalate as a Ag precursor, a smaller carboxylate to citrate, detailing that it was toxic, unstable and potentially explosive, opted to use it just a few months later,^[34]^ due to its low decomposition temperature and high Ag content. Yang and co‐workers produced a silver oxalate‐based ink dissolved in ethanol, ethylene glycol and butylamine, again producing an ink with around 10 wt.% Ag. Again, the ink was drop‐casted onto PI and sintered under various temperatures (110–160 °C, chosen due to the findings of DSC and UV/Vis analysis of the ink) and times up to 1 h. The lowest resistivity was again achieved at the highest sintering temperature, producing a film with *ρ*=8.19×10^−8^ Ω m, around ten times better than the silver citrate ink sintered at 155 °C (*ρ*=7.94×10^−7^ Ω m).^[33]^

Hu et al. synthesised a particle free transparent conductive ink involving the rare use of fluorinated precursor in a MOD ink in the form of silver trifluoroacetate.^[35]^ The ink was synthesised by dissolving a small amount of salt in butanone, followed by the addition of polystyrene‐block‐polyisoprene‐block‐polystyrene (SIS). Small amounts of the high BP solvent dimethylacetamide (DMAc) were added to the ink which improved its fluidity and prevented clogging during deposition—a typical 9:1 volume ratio of butanone:DMAc was used. Patterns were handwritten onto SIS substrates using a ballpoint pen filled with fresh ink. After, the written deposits were reduced using a mixed solution of formaldehyde and sodium hydroxide (NaOH) for 5 min, to make them conductive. Typically, the conductivity was inadequate when one‐time writing was undertaken due to the thinness of the Ag layer, however a low line resistance (80.03 Ω m^−1^) from the written trace of the pen of could be achieved after repeated patterning and in situ reduction cycles. After a strain of 100 % the line resistance increased by over ten‐fold to 1040 Ω m^−1^, with authors stating this value was still sufficient for the function of common electronic devices.

Taking molecular structure into account Black et al. used a different type of Ag precursor from the rest,^[36]^ namely a fluorinated β‐diketonate Ag precursor. Inspired by the Ag atomic layer deposition field, Black et al. used the metal‐organic compound: [(hfac)(1,5‐COD)Ag] (where hfac=hexafluoroacetylacetone) in their MOD ink. Acetylacetones^[37]^ and their fluorinated counterparts are commonly found in vapour deposition techniques of metals and metal oxide,[^38^, ^39^] but are rarely used in MOD ink chemistry. The hfac compound was dissolved in anhydrous toluene followed by the addition of isopropanol under inert conditions, producing a series of inks with varying organometallic/alcohol ink ratios. The inks were inkjet printed onto pre‐heated glass substrates (90–120 °C) under ambient conditions. The least resistive metal film (*ρ*=4.10×10^−8^ Ω m, ×2.5 that of bulk Ag) was deposited using a 0.5 m:0.5 m organometallic‐alcohol ink formation at 120 °C.

From these recent contributions to the literature, it is evident that low resistivity targets are met, often at the cost of requiring longer sintering times. Decomposition temperatures vary, dependent on substrate, ink formulation and sintering method which suggests potential for Ag MOD inks to be ‘made‐to‐measure’ for the task at hand. Shelf life and adhesion properties are evidently hot topics in this area and continue to improve.

#### 3.1.2 Silver NP inks

Ag NP inks are the most researched type of all conductive inks, with over one hundred articles in this field over the last decade. Ag NP inks also dominate industrially, with many currently available commercially. In this Minireview we have curated a selection of the most scientifically important investigations, with research that uses commercial NPs and/or inks omitted.

Due to Ag's high resistance to oxidation, the biggest concerns with regards to designing a successful Ag NP ink are its concentration, stability in solution, sintering temperature and rheological properties. Unquestionably, the most common method over the last decade employed to optimise such variables are capping agents. Capping agents are key in tailoring NP properties such as dispersion and stability within a solvent, which affects Ag NP saturation concentrations. Several variables such as capping agent chain length, whether mixed capping agent systems are used, and the capping agent solvent interaction can be used to produce NP inks with desirable rheological and stability properties. NP and capping agent interactions determine the temperature at which conductive films can be produced, with the strength of binding mainly determined by the functional group on the capping molecule. A balance must be achieved between the activation energy for decapsulation and energy barrier to conversion to prevent unstable encapsulation, while simultaneously ensuring low conversion temperatures of the capped NPs to conductive metal. Non‐conventional methods of deposition/sintering/annealing can also be key in lowering the deposition temperatures/times of inks containing capped NPs.

**Figure 4:**
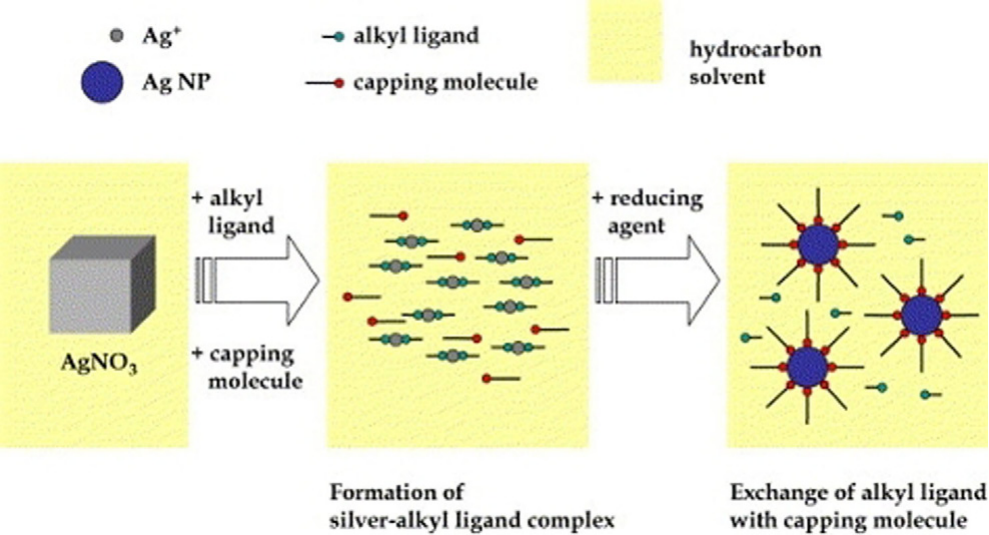
Experimental schemes for direct synthesis of Ag NPs in fully organic phase through in situ ligand exchange. Reproduced with permission from ref. [40], Copyright 2006, Elsevier.

A common encapsulation procedure is detailed by Lee et al.;^[40]^ a Ag salt (most commonly AgNO_3_) is dissolved in a solution of alkyl ligand (e.g., amines) and a solvent medium (i.e., toluene) forming a silver‐alkyl ligand complex. Capping molecules (amines, acids, polymers etc.) are subsequently added to the solution before the silver‐alkyl ligand complexes are reacted with a reducing agent (such as sodium borohydride (NaBH_4_) or hydrazine) resulting in NP formation and exchange of the alkyl ligand with capping molecules (Figure 4).

Based on a study by Peng et al. which showed amine functional groups exhibited higher binding energies than carboxylic acids to low‐index Ag surfaces with the same chain length,^[41]^ Ankireddy and co‐workers employed a series of short chain carboxylic acids (C6‐C10) to encapsulate Ag NPs,^[42]^ in order to produce inks that could deposit conductive metal films at lower temperatures, whilst still maintaining good stability and rheological properties for direct‐write systems. Capped NPs were synthesised using a modified synthesis by Lee et al.,^[40]^ with capped NPs dispersed in toluene for all ink formulations. The loading of Ag NPs was increased with increasing capping agent chain length, producing a series of inks with concentrations ranging from ≈3—66 wt.%, with most inks being stable for a minimum of 1 month. As ink concentration increased sheer thinning behaviour became more prominent, surface tension decreased and the contact angles of the inks on PI and glass substrates increased. Microelectrodes were fabricated via aerosol jet printing and ultrasonic spray coating, producing features with conductivities 10–87 % of bulk Ag *(ρ*=1.59×10^−7^ Ω m–1.83×10^−8^ Ω m), with sintering temperatures ranging from 130–250 °C. The authors were hesitant to draw direct correlations between encapsulant chain length and the performance of their corresponding NP ink formulations, due to the large range of variables.

**Figure 5:**
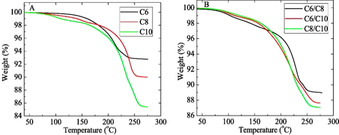
(a) TGA curves for C6, C8, and C10 particles. (b) TGA curves for C6/C8, C6/C10, and C8/C10 particles. Reproduced with permission from ref. [43], Copyright 2013, AIP.

Later, Ankireddy et al. extended their investigation into mixed carboxylic acid capped NP systems,^[43]^ and found that the decomposition temperature for mixed encapsulant Ag NPs were close to the average of the corresponding decomposition temperature of single encapsulant NPs. A more systematic study enabled the authors to confidently state that for singular encapsulant systems, increased chain length led to increased NP decomposition temperature, which was investigated by thermogravimetric analysis (TGA) (Figure 5). As proof of concept, Ankireddy and co‐workers fabricated a conductive antenna on PI using an ink with C10 capped NPs (*ρ*=3.38×10^−8^ Ω m, 240 °C for 1 h).

Other larger carboxyl containing compounds have also been used to encapsulate Ag NPs, with great success, for example, oleic acid. Jo et al. exhibited that their oleic acid encapsulated Ag NP ink showed great potential towards adapting to roll‐to‐roll processing.^[44]^ Capped NPs were synthesised by reducing a AgNO_3_/octylamine/oleic acid solution heated to 80 °C with phenylhydrazine. After work‐up, NPs were dispersed in a high molecular weight block copolymer and toluene, producing a 20 wt.% ink. Conventional thermal sintering of films was undertaken between 100–300 °C, but long sintering times were required, so instead the inks were subjected to an “instant photonic sintering process ”. With printed films dried in air for 10 min prior to sintering, Ag metal films were formed on a timescale of 1.0–1.5 ms at 2–3 kV photon energy, showing *ρ*=5.00–8.00×10^−8^ Ω m on PI and PET substrates. Metallic films also exhibited good durability and flexibility, with films on plastic tested over 10 000 cycles with a bending radius of 1.5 mm.

**Figure 6:**
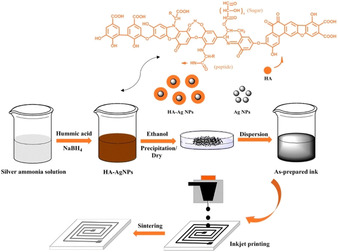
Schematic illustration of the preparation of HA‐Ag NPs and printed patterns from Hao et al. Reproduced with permission from ref. [45], Copyright 2019, Springer.

More recently in 2019, Hao et al. used the naturally occurring humic acid to stabilise Ag NPs via chelation through a series of O and N functional groups.^[45]^ Capped NPs were synthesised via the reaction of silver ammonia with aqueous humic acid solution, after which the Ag^I^ was reduced by NaBH_4_ (Figure 6). The humic acid capped NPs were washed, filtered, and dried before being dissolved in ultrapure water, with isopropanol added to modify viscosity and surface tension. TGA showed that the Ag content of the capped NPs measured as high as 75 wt.%, whilst the best performing ink in terms of stability (>30 days) and conductivity was the 2.5 wt.% ink. The 2.5 wt.% conductive ink was printed onto photopaper to fabricate conductive Ag patterns with a domestic inkjet printer. The best *ρ*=1.35×10^−6^ Ω m was measured after printing 40 layers and sintering at 180 °C for 1 h.

Shankar et al. used amines as an alternate capping agent to carboxylic acids,^[46]^ with dodecylamine encapsulated Ag NPs synthesised by reducing silver acetate in an amine‐toluene solution using tin acetate. The resulting small particles (5–20 nm) were dissolved in non‐aqueous solvents to produce a 20 wt.% conductive ink, which was used to print conductive tracks on flexible substrates (photopaper, PI) using an aerosol jet‐based printing technique. Printed Ag tracks were sintered between 180–300 °C, with the most conductive films formed unsurprisingly at higher temperature (*ρ*=≈1.59×10^−7^ Ω m).

Yamada et al. investigated a range of amines for use as encapsulants for Ag NPs in conductive inks.^[47]^ Ag NPs were synthesised from the thermal decomposition of silver oxalate as detailed by Itoh et al.,^[48]^ in the presence of amines (typically a mixture of *N*,*N*‐dimethyldiaminopropane, hexylamine and dodecylamine) at 110 °C in the presence of a small amount of oleic acid. Ag nanometal inks were prepared in the concentration range between 40–60 wt.% using *n*‐octane and *n*‐butanol as solvents. The deposition process involved the chemisorption of weakly encapsulated Ag NPs onto a photoactivated surface. UV light was used to irradiate a perfluorinated polymer layer to photoactivate the surface with pendant carboxylate groups, with subsequent blade coating of the amine encapsulated Ag NPs, which causes “amine‐carboxylate conversion ” to trigger the spontaneous formation of continuous Ag coatings. The printed Ag layers dried in vacuum exhibited decent conductivities of *ρ*=≈1.00×10^−6^ Ω m with no post‐treatment, whilst waiting longer durations of time and/or by annealing at low temperature (<80 °C) conductivities were improved by up to an order of magnitude.

Ghosale et al. also used amines to encapsulate NPs,^[49]^ synthesising octylamine capped NPs following a method by Tobita et al.,^[50]^ which used ascorbic acid to reduce a solution of AgNO_3_ and amine in toluene. After work‐up, NPs were dissolved in chloroform to make a 10 wt.% ink, which was used to fabricate Ag electrodes on paper (via direct‐writing using a ball‐point pen) for the detection of hydrogen peroxide in wastewater samples, showing the versatility of conductive metal tracks. Optimised sintering conditions of 100 °C for 1 h were used to make the electrodes conductive (*ρ*=3.06×10^−7^ Ω m).

Whilst this Minireview has highlighted some interesting recent reports of using discrete carboxylic acid and amine capping molecules, unquestionably the most common family of capping agents in the Ag NP literature are polymeric, with most of them containing carboxyl terminal groups. A good example of this was presented by Shen et al.,^[51]^ who synthesised polyacrylic acid (PAA) capped Ag NPs, using AgNO_3_, and ethanolamine as the reducing agent. Ag inks with concentrations of 5—25 wt.% were formulated using deionised water as the solvent, forming highly stable dispersions, with good homogeneity. Surface tensions, densities, viscosities, and printability were calculated for all ink concentrations. The 20 wt.% ink was then used in several experiments that varied annealing temperature and the number of printing cycles, depositing onto photo‐paper and PET substrates, using a standard printer. Following air drying for 1 day, annealing the films at 180 °C, the highest temperature used in this study, yielded Ag films with *ρ*=3.70×10^−8^ Ω m, just over twice as resistive as that of bulk Ag. LED device circuits were fabricated using inkjet printing and sintering at low temperature (<100 °C) to validate the potential applications of such inks for use in the fabrication of flexible electronics. In 2018 Shao et al. produced large Ag NPs (50–70 nm) by the facile reduction of AgNO_3_ with hydrazine hydrate in the presence of PAA and ethanolamine.^[52]^ PAA‐alkanolamine capped NPs were synthesised and then formulated into an ink by dispersion in an ethylene glycol butyl ether, ethylene glycol, hydroxyethyl cellulose mixed solvent system, along with other additives that are not explicitly stated. TGA showed the NPs were approximately 97.5 wt.% Ag. The Ag‐based conductive ink was deposited onto PI substrates under differing temperatures using their “rapid thermal annealing system ”, with temperatures as low as 150 °C used. Films annealed at >200 °C showed very low resistivities (*ρ*=5.60×10^−8^ Ω m at 250 °C for 1 h), ×3.5 that of bulk Ag.

In the same year Huang et al. synthesised polydisperse Ag NPs with diameters ranging from 5–85 nm,^[53]^ also using PAA as the stabilising agent. Interestingly, NPs were sintered in the presence of a chloride (NaCl), a method of chemical sintering that has been reported to reach low temperatures (80 °C).^[54]^ An AgNO_3_ aqueous solution was reduced by a combination of PAA and triethanolamine to produce the silver NPs, whilst the ink solvent consisted of a NaCl solution, propylene glycol and carboxymethylcellulose sodium, which acted as the binding and thickening agent; producing a 47 wt.% ink. Films were deposited onto PET via screen printing with an exemplar result of a Ag metal film with *ρ=*1.52×10^−7^ Ω m after sintering at 120 °C for 1 h.

In 2019, arguably at the cost of higher temperatures and times Lee et al. printed moderately conductive (*ρ*<10^−6^ Ω m) patterns using 3D printing.^[55]^ A low viscosity (≈7 MPa s) 25 wt.% PAA capped Ag aqueous NP ink produced several conductive 3D patterns with diameters of a few micrometres, which were sintered at 250 °C for 1 h. Synthesis of NPs and ink formulations is similar to that found in the literature for when PAA capped NPs are used as the capping agent, however in this method microwave radiation was used to assist synthesis.

Hao et al. also used polymers to stabilise Ag NPs,^[56]^ using the carboxyl‐terminated hyperbranched polyester (CHBP). CHBP was added to a silver ammonia solution, whilst aqueous ammonia was used to maintain a pH of 9–10. The Ag solution was reduced using NaBH_4_, forming uniform and monodisperse capped NPs, with a diameter of ≈10–20 nm, after work‐up. The obtained CHBP‐Ag NPs could be kept in their powder form for several months, making them convenient for transportation and storage. CHBP‐Ag NPs aqueous inks (5—15 wt.%) were obtained by merely dispersing CHBP‐Ag NP solid powder into ultrapure water—all of which showed good stability after 30 days. The authors report a conductive pattern that had 30 printed layers and was sintered at 180 °C for 1 h 20 min, producing a film with *ρ*=1.08×10^−7^ Ω m, just under seven times that of bulk Ag.

**Figure 7:**
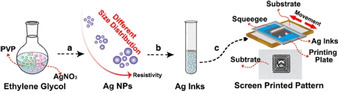
Schematic illustration of the preparation of Ag NPs based conductive inks and conductive patterns. a) the controlled mass ratio of AgNO_3_ and PVP for obtaining Ag NPs with different size distribution. b) the as synthesized Ag NPs are dispersed into ethanol for the formation of Ag inks. c) screen printing of Ag inks on different substrates. Reproduced with permission from ref. [57], Copyright 2016, Springer.

Non‐carboxyl terminated polymers have also been used to encapsulate Ag NPs for use in conductive inks such as the work by Ding et al.,^[57]^ who employed a facile one‐step polyol method to synthesise polyvinylpyrrolidone (PVP) capped Ag NPs on a large scale. Changing the mass ratio of AgNO_3_ and PVP, resulted in different average NP diameters (52–120 nm) and particle distribution. Additionally, the as‐obtained Ag NPs were prepared as ≈70 wt.% Ag conductive inks, which were screen printed on various flexible substrates with the results of electronic properties revealing that the conductivity was highly dependent on the size distribution of as‐obtained Ag NPs (Figure 7).

The optimal conductivity was obtained when samples utilised a mass ratio of AgNO_3_ and PVP of 1:0.4. The authors subsequently investigated the effects of sintering time and temperature towards obtaining metal films with the best conductivity—an optimal *ρ*=3.83×10^−8^ Ω m achieved at 160 °C sintering for 1 h 15 min, just over twice the resistivity of bulk Ag.

Fernandes et al. also synthesised PVP capped Ag NPs using a more conventional method—reducing AgNO_3_ with NaBH_4_ in the presence of PVP.^[58]^ NPs were dissolved in various solvents to make up solutions with weight loadings of either 8 or 16 wt.%, with ethylene glycol, ethanolamine and small amounts of polyesters. All inks had a viscosity between 3.69–7.41 MPa s, facilitating inkjet printing. Inks were then thermally sintered at either 150, 200 or 300 °C, with the latter producing films with the best resistivity, as expected. From the seven inks produced, there was not a clear best candidate. For example, the ink that displayed the best electrical properties after 150 °C sintering (*ρ*=5.40×10^−7^ Ω m), was not the best ink when sintered at 300 °C (*ρ=*5.60×10^−8^ Ω m) and neither were the most stable. The most stable inks contained the humectants and dispersants, with two such 16 wt.% inks used to deposit Ag metal for electrodes.

Finally, in addition to molecular and polymeric methods, sodium citrate salts can also be used to encapsulate Ag NPs. Recently, Yin et al. showed using thermal analysis that the onset of decomposition for sodium citrate capped NPs was 110 °C. As such, a 20 wt.% Ag content ink was used to deposit conductive Ag metal films, with a best *ρ=*1.56×10^−7^ Ω m when sintered at 180 °C for 20 min.^[59]^ Ranoszek‐Soliwoda et al. also used sodium citrate to encapsulate Ag NPs,^[60]^ however an additional capping agent, tannic acid, was also used. It was reported that the combination of the two capping agents controlled nucleation, growth and stabilisation, producing monodisperse spherical NPs.

**Figure 8:**
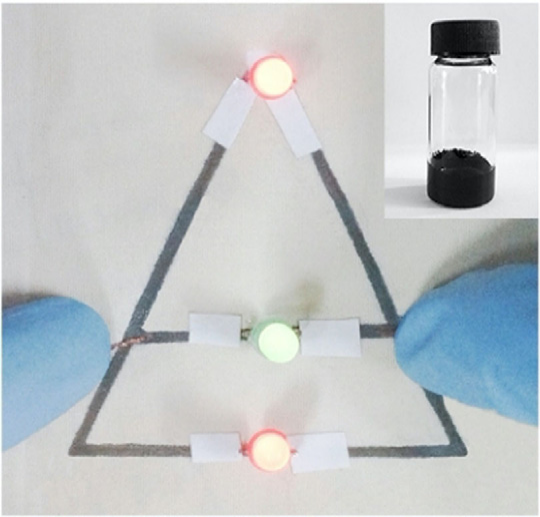
The photo of Wang et al.’s silver NP ink printed on cotton. Reproduced with permission from ref. [63], Copyright 2016, Elsevier.

Sodium citrates can also be used as reductants, as opposed to capping agents, especially in the presence of ferrous sulphate which forms the strong reductant ferrous citrate in situ, as first utilised by Carey Lea.^[61]^ Kardarian et al. used a novel in situ synthesis of Ag NPs at the textile fibre surface,^[62]^ followed by sintering to obtain moderately conductive fabrics. Samples of cotton fabric were immersed in an aqueous AgNO_3_ solution with heating under constant stirring. During heating, NaOH was added until no further precipitation of Ag occurred. Upon boiling of the solution, an aqueous trisodium citrate solution was also added to aid metal reduction, before the cotton fabric samples were washed. Epoxy‐silane was used before the washing process to improve binding of the Ag NPs to the fabric, requiring curing temperature of 140 °C for 20 min. The robustness of the Ag NP fabrics was tested by washing various samples with detergent. Fabric samples were sintered over a range of temperatures and times with a best *R_S_* of 5.20 Ω sq^−1^, achieved under sintering conditions of 200 °C for 30 min. Similar work to this was reported in 2016; Wang et al. also deposited Ag onto cotton fabric,^[63]^ using a 30 wt.% ink of 10 nm NP's synthesised via an “improved ” Carey Lea method. Circuits were screen printed onto the cotton substrate followed by a spontaneous coalescence and sintering process of Ag NPs at 60 °C for 30 min in the presence of a hydrogen chloride (HCl) catalyst. The most conductive Ag metal films were deposited when the HCl concentration was 50 mm (polyaniline 27.8 wt.%), with a modest *ρ*=2.00×10^−5^ Ω m on cotton (Figure 8).

The use of chloride anion sources such as NaCl, MgCl_2_ and HCl as Ag NP sintering agents likely derives from the work in the 2010s from the Magdassi group.[^54^, ^64^] Grouchko et al. presented a Ag NP‐based conductive ink,^[54]^ that had “a built‐in sintering mechanism ”, which is triggered during drying of the printed pattern. The polymer encapsulated NPs undergo self‐sintering spontaneously, due to the presence of a destabilising agent in the form of a Cl^−^ ions, which comes into action only during drying of the printed pattern. Exposing their inkjet printed metallic films to HCl vapours for 10 s, produced films with superior conductivities compared to when NaCl was used. Films with conductivity 41 % of that of bulk of Ag were produced at RT (*ρ*=3.84×10^−8^ Ω m).

Other synthetic methods to improve Ag metal film deposition at low temperature than just altering capping agents have been reported, by Liu et al. in 2018.^[65]^ This study investigated the effect of bimodal inks composed of two sizes of Ag NPs (10 and 50 nm). The Ag NPs were synthesised using a modified reduction method based on that by Carey Lea,^[61]^ with the synthesis of the larger 50 nm NPs requiring a higher temperature, a lower AgNO_3_ solution concentration and the absence of ferrous sulphate, for a slower reduction process. All inks were printed and sintered at RT on photopaper without chemical treatment. An excellent *ρ=*3.66×10^−8^ Ω m film was obtained after 17 inkjet printing cycles (>1 min drying per step), when the ratio of the 10 nm Ag NPs to the 50 nm Ag NPs was 2:1—films could be printed in a short time, exhibited better bending performance and had reduced coffee‐ring defects compared to their unimodal counterparts. The smaller 10 nm particles when sintered favoured the formation of necks for electrical contacts, whilst the larger 50 nm particles allowed for the formation of large, sintered cells which acted as infrastructure for improved connectivity.

Finally and untypically, Murtaza et al. synthesised Ag NPs via a ligand replacement reaction between a very low concentration (0.002 %) aqueous AgNO_3_ solution by jet propulsion through a solid Cu tube.^[66]^ After work‐up, the Ag powder obtained was used to formulate a 35 wt.% ink using ethanol as the solvent and polyvinyl bromide as the binder and also to protect against oxidation. The conductive ink was direct written onto paper substrates, producing the most conductive metallic film (*ρ=*6.70×10^−8^ Ω m) when annealed at 110 °C for 5 h. Deposited films exhibited a small loss of conductance after 2000 bending cycles (1.95 %) and after storing the ink for 20 days at 80 °C (1.29 %), indicating a limited shelf life.

It is evident from these reports that undoubtedly larger capping agents will result in a temperature/time compromise. Successful printing from a range of capping agents, from amines, through acids, polymers and even reactive NPs with reductive salts can all form highly conductive coatings. Shelf life and adhesion properties are not prioritised as highly as printer precision, when compared to the MOD literature which is interesting. Excellent results are reported on the mixing of NP sizes, which would seem an obvious direction for future investigations.

### 3.2 Copper inks

Owing to its position in the periodic table Cu is the second most conductive element (*ρ*=1.72×10^−8^ Ω m). In contrast to Ag, many reports of Cu deposition are carried out in an inert environment due to its increased reactivity toward oxygen resulting in oxide formation. However, it is still heavily researched, with literature contributions on the topic of Cu NP inks coming second in volume only to Ag. Cu boasts superior resistance to electromigration compared to Ag in its printed tracks or coatings and is also one hundredth of the price, making its less trivial deposition conditions viable.

#### 3.2.1 Copper MOD inks

Cu precursors, or MOD compounds, can be synthesised from a variety of routes (Scheme 1) and are distinct from NPs as in these molecules they exist as oxidised centres, and not in the metallic ‘zero’ oxidation state. Owing to Cu^I^’s tendency to disproportionate to Cu^0^ and Cu^II^, Cu^II^ MOD inks dominate the research. In these inks, where Cu exists in its most oxidised state, the problem of oxidation during storage is solved, since it cannot oxidise any further.

**Figure 9:**
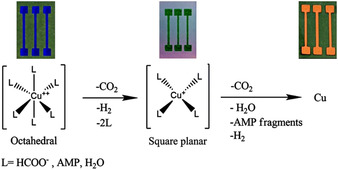
Farraj et al. explain their MOD decomposition pathway. Reproduced with permission from ref. [15], Copyright 2015, Royal Society of Chemistry.

At the end of the last century, Galeway et al. conducted extensive research into the thermal decomposition properties of a number of Cu salts; like Cu^II^ oxalate,^[67]^ Cu^II^ maleate and Cu^II^ fumarate,^[68]^ Cu^II^ squarate,^[69]^ and Cu^II^ formate (Cuf).[^70^, ^71^] However, early research into the use of Cu^II^ salts in inks focused on Cu‐β‐diketonates, β‐ketoiminates and β‐ketoesters. Following this, in the last decade, nearly all research has been focused on Cuf and its derivatives due to several reasons. Firstly, after the formate anions oxidise during decomposition producing Cu metal, the by‐products (hydrogen gas (H_2_), carbon dioxide (CO_2_) and formic acid (HCOOH)) provide a reducing atmosphere. This helps prevent Cu re‐oxidation, and negates the need for harmful external reducing atmospheres (such as H_2_), making them more suitable for industry. Cu^II^ compounds decompose to Cu metal via a Cu^I^ intermediate, which is accompanied by a striking colour change from blue, to green and finally to a pinkish‐orange lustre of the metal (Figure 9).^[15]^

**Figure 10:**
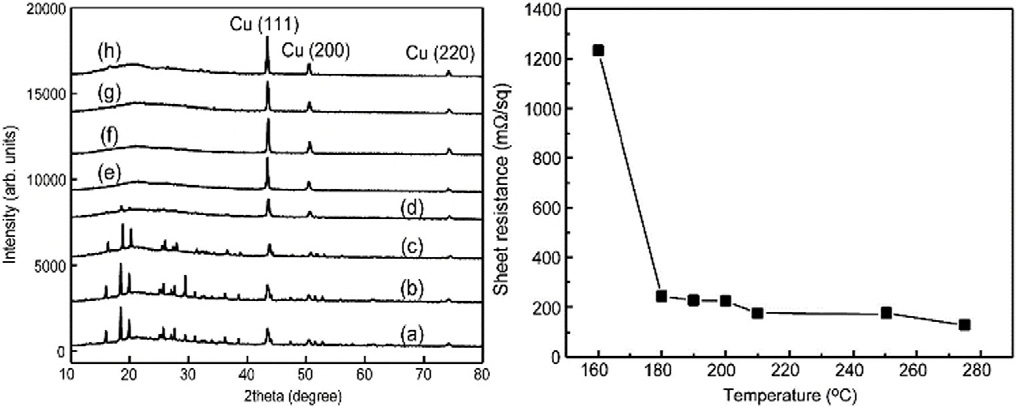
Left: XRD patterns of films annealed at different temperatures. (a) as printed, (b) 100 °C, (c) 120 °C, (d) 140 °C, (e) 160 °C, (f) 180 °C, (g) 200 °C, (h) 275 °C. All unlabelled peaks are from the unconverted Cuf precursor. Right: variation of *R_s_* as a function of the annealing temperature. Reproduced with permission from ref. [73], Copyright 2012, Royal Society of Chemistry.

Between 2010–2015, the viability of Cuf in various solvents with different additives was assessed by multiple research groups. Joo et al. synthesised a Cu paste with 76.2 wt.% Cu,^[72]^ by dissolving Cuf in α‐terpineol with the addition of a dispersion agent. Laser sintering under nitrogen gas (N_2_) on a PI substrate yielded a Cu film with *ρ*=1.86×10^−7^ Ω m. They subsequently used the paste to compare different sintering methods on PI and found that thermal sintering at 275 °C for 1 h resulted in a lower *ρ*=1.30×10^−7^ Ω m (>10 times bulk Cu), compared to the laser sintered film (*ρ*=1.41×10^−7^ Ω m) for the same duration of time. This work also included an excellent example of how MOD ink conversion can be monitored using XRD (Figure 10, left)—as the sintering temperature increased the precursor peaks decreased until only Cu metal was detected. Additionally, the inverse relationship between resistance and sintering temperature that is common to all metal deposition was observed (Figure 10, right).^[73]^

Later in 2012, Wang et al. used a Cuf ink with PVP, ethylene glycol and 2‐methoxyethanol as additives.^[74]^ Substrates were treated by a N_2_ plasma; to improve adhesion and mechanical properties of the film. Laser sintering in air (without the presence of a reducing or inert atmosphere) produced a low *ρ=*4.62×10^−8^ Ω m film on PI and PET substrates. Optimisation of the laser energy proved crucial, as at lower energy values the organics were not completely incinerated, preventing sintering of the Cu particulates resulting in higher resistivity. Interestingly, at higher laser power and speed, a steady production of shielding by‐product gas was not produced. This resulted in oxidation of the forming Cu particulates and increased their resistivity. Rosen et al. used a Cuf dispersion in distilled water (21 wt.% Cu).^[75]^ Using a reactive transfer printing method and heating under a N_2_ environment, they obtained conductive Cu films on glass with the lowest *ρ*=3.29×10^−8^ Ω m obtained when heated at 200 °C for 30 min. Using the same ink in combination with single walled carbon nanotubes (SWCNT) and PVP additives, an intense pulsed light (IPL) was applied to obtain films with resistance of 9.62 Ω on a PI substrate. However, to obtain conductive films the printed dispersion was dried at 70 °C for 5 min to remove the solvents.^[76]^ The addition of the SWCNT reduces the required threshold energy of the laser which helped prevent damage to the temperature sensitive substrate.

While Cuf inks proved successful in depositing conductive films, their solubility in more common organic solvents is limited thereby creating a hindrance for use in various printing techniques. To overcome this, a Cuf ink complexed with *n*‐octylamine was synthesised by Yabuki et al. in 2011.^[77]^ TGA of pure Cuf, and Cuf complexed with *n*‐octylamine revealed that bonding with the amine groups exhibited a markedly lower decomposition temperature than pure Cuf. Printed patterns on glass first dried at RT for 30 min followed by sintering at 140 °C for 1 h, under a N_2_ environment resulted in conductive films with *ρ=*2.00×10^−7^ Ω m.

Yabuki's work spurred great interest in inks formed by complexation of Cuf with various organic ligands as it not only allowed Cuf's dissolution in non‐aqueous solvents, but it could also result in lower complex decomposition temperatures. Yabuki et al. later tested the effect various amines have on the deposition properties of the film by complexing Cuf with various primary and secondary amines,^[78]^ with the effects of complexing Cuf with a blend of different amines also investigated. The chain length and BP of the amines largely affected the resistivity of the deposited films. Primary amines resulted in the formation of smaller particulates with dense packing, with the particulate size and density increasing with carbon chain length. Shorter secondary amines resulted in loosely packed larger particulates, resulting in voids that increased resistivity. To optimize the properties of the ink, the lower BP dibutylamine (20 mol %) was blended with *n*‐octylamine to resulting in a densely packed film on glass with *ρ=*5.00×10^−8^ Ω m, (under N_2_ at 140 °C for 30 min).

**Figure 11:**
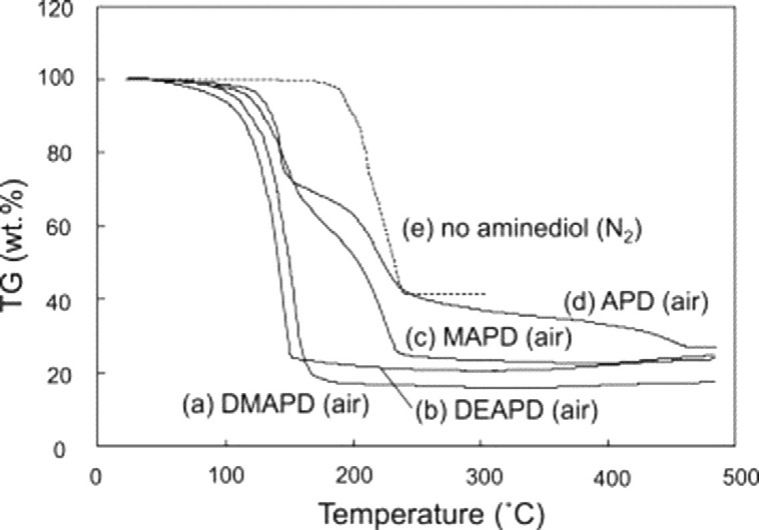
Yabuki et al.’s thermal analysis evidenced a marked decrease in decomposition temperature upon ligand coordination: (a) 3‐dimethylamino‐1,2‐propanediol, (b) 3‐diethylamino‐1,2‐propanediol, (c) 3‐methylamino‐1,2‐propanediol, (d) 3‐amino‐1,2‐propanediol, (e) no aminediol. Reproduced with permission from ref. [80], Copyright 2014, Elsevier.

Shin et al. utilized alkanolamines as ligands to synthesise Cu MOD inks.^[79]^ The presence of the alkanolamine ligands not only helped increase complex solubility in alcohols but also resulted in a lower decomposition temperature compared with uncomplexed Cuf. While various primary and secondary alkanolamines were studied, the tertiary 2‐amino‐2‐methyl‐1‐propanol (AMP) showed superior performance, which is attributed to the presence of the tertiary carbon and increased steric hindrance which prevents the AMP from undergoing carbamate polymerisation, thus reducing the amount of organic residues present in the sintered film, compared to the primary and secondary alkanolamines. The film obtained from the Cuf‐AMP ink unfortunately showed large voids under SEM analysis, so to overcome this, octylamine was added to control particulate size and hexanoic acid was added to improve sintering. This resulted in films with *ρ*=2.34×10^−7^ Ω m when sintered at 200 °C for 30 min on glass under a N_2_ environment, which decreased to *ρ*=9.46×10^−8^ Ω m (×5.5 that of bulk Cu) when sintered at a higher temperature of 350 °C. Yabuki et al. also used dihydroxy‐alkanolaminedihydroxy‐alkanolamines as the complexing agent to determine their feasibility for use as inks.^[80]^ Promisingly, decomposition temperatures of the 3‐dimethylamino‐1,2‐propanediol (DMAPD) and 3‐diethylamino‐1,2‐propanediol (DEAPD) Cuf complexes was found to be lower than pure Cuf (Figure 11). Moreover, the dihydroxy‐alkanolamines adsorbed onto the surface of Cu particulates during sintering helping prevent the oxidation of the Cu particulates and thus, films could be sintered in air. The DEAPD complexed with Cuf ink when sintered in air at 180 °C for 5 min resulted in a moderately conductive Cu film on glass with *ρ=*3.00×10^−6^ Ω m. Farraj et al. also used a Cuf‐AMP complex, comparing the films obtained using anhydrous and dihydrate Cuf as the reagent.^[15]^ Somewhat surprisingly, it was reported that films deposited using the hydrated Cuf had lower resistivity than those deposited using inks formed with anhydrous Cuf (*ρ*=1.05×10^−7^ Ω m and 4.50×10^−7^ Ω m, respectively when heated at 190 °C under N_2_), improving on the above mentioned work reported by Shin et al.^[79]^ Two possibilities were presented for this: the water could either be acting as a catalyst for Cuf decomposition or it could be acting as an acid, due to its amphoteric nature, that assists decomposition of the basic ligands.

Xu et al. studied the effect that amine chain lengths had on the deposited Cu films.^[14]^ Various primary amines with differing chain lengths were complexed with anhydrous Cuf. Longer chain amines decomposed slower than shorter chain ones. The undecomposed amines capped the Cu particulates being formed and helped regulate particulate size resulting in dense, uniform films (as evidenced by SEM images). Consequently, the resistivity of the films decreased as amine chain length increased. However, longer chain lengths also resulted in higher amounts of organic residue remaining in the film post sintering at low temperatures, which were at a detriment to film performance. SEM revealed that short chain amines, because of their quick decomposition resulted in a wide size distribution of Cu particulates and left the Cu particulates susceptible to oxidation however, they could easily be removed from the films at low temperatures with little organic contaminants outstanding. Therefore, it was decided in order to achieve optimal film performance, a 50:50 blend of short chained (butylamine) and long chained (octylamine) complexing amines should be used. While the octylamine provided a good capping effect, replacing 50 % of it with butylamine resulted in reduced organic residues and a best film with *ρ=*4.28×10^−8^ Ω m was obtained on glass when sintered at 200 °C for 40 min. Conductive films, with resistivities five times that of bulk Cu, using this ink were also obtained on the more flexible PI (*ρ*=9.69×10^−8^ Ω m; 180 °C for 20 min), PET and PEN substrates (*ρ*=2.29 and 2.14×10^−7^ Ω m, respectively; 160 °C for 20 min).

**Figure 12:**
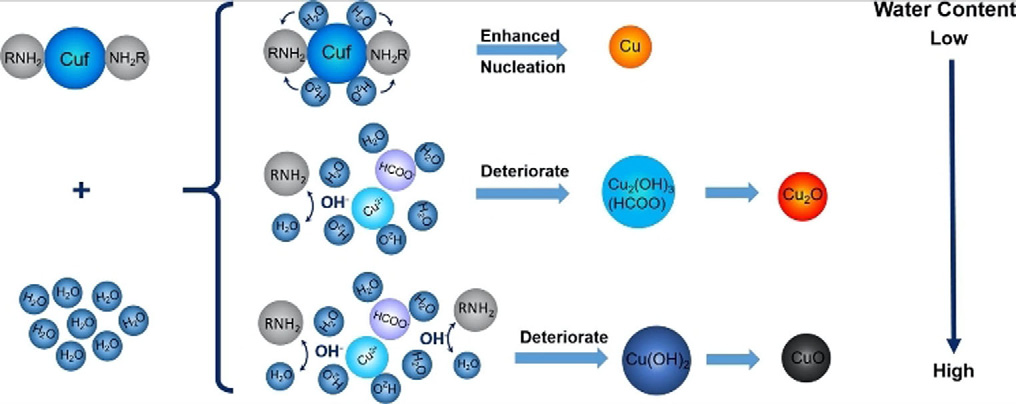
The effect of water on MOD decomposition. Reproduced with permission from ref. [82], Copyright 2018, Elsevier.

Huang et al. synthesised a Cuf ink complexed with mono‐isopropanolamine.^[81]^ Octylamine was added to the ink to improve surface tension and PVP was added as an adhesion promoter. Thermal sintering of the ink on glass at 140 °C for 5 min under N_2_ resulted in films with *ρ=*2.00×10^−7^ Ω m. Following this in 2018, Xu et al. aimed to optimise Cuf based inks by studying the effect of water concentration on shelf life and film resistivity (Figure 12).^[82]^ By varying the coordinating ligand and the amount of water in the inks, they found that the 95:5 blend of 2‐ethylhexylamine and AMP ink with 2 wt.% water exhibited the best shelf life. Films with *ρ=*5.20×10^−8^ Ω m were obtained when heated at 250 °C for 30 min on PI substrates, just under three times that of bulk Cu. Dong et al. were able to use their Cuf complexed with 1,2‐diaminepropane ink to obtain films with *ρ=*1.80×10^−7^ Ω m on PI when heated at 180 °C for 1 min when sintering in air.^[83]^

More recently, Yabuki et al. studied the decomposition profiles of inks synthesised by complexing Cuf with various low BP amines (butylamine, pentylamine and octylamine).^[84]^ They deposited conductive Cu films on glass by sintering the inks near their onset temperatures for decomposition, with the Cuf‐pentylamine ink performing best. By varying the molar ratio of pentylamine:Cuf and looking at the effect this has on the size of the deposited particulates and thus, the resistivity, they were able to obtain a film with *ρ=*5.70×10^−8^ Ω m (three time that of bulk copper) on glass. This was obtained for a pentylamine:Cuf molar ratio of 2:4, when thermally sintered at 110 °C for 30 min under a flow of N_2_.

The decomposition temperatures of pyridine complexed Cuf have been found to be lower than those where alkylamines were used as ligands. However, due to the lower surface tension of pyridines, films formed using these resulted in a lot of cracks which has deterred the use of pyridines in the industry. In an effort to overcome this, Paquet et al. used a 60:40 blend of 3‐butylpyridine and 2‐ethyl‐1‐hexylamine.^[85]^ Films with *ρ*=6.50×10^−8^ Ω m on PEN were obtained when heated at 170 °C for <5 min under N_2_.^[85]^

**Figure 13:**
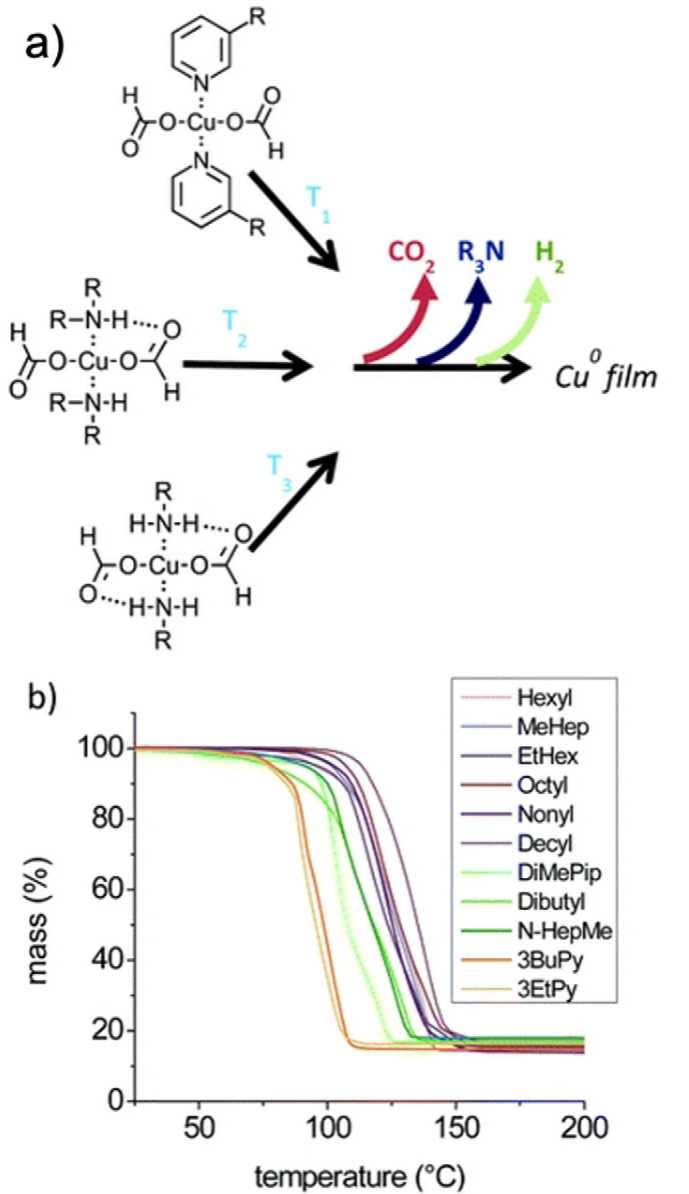
Paquet's study revealing a) The temperature at which Cuf complexes begin to decompose is governed by the number of hydrogen bonds that can form between the amine hydrogen and the formate oxygen, b) TGA of Cuf coordinated to various amine ligands. For clarity, complexes composed of primary amines have purple TGA curves, secondary amines have green TGA curves and pyridines have orange TGA curves. Adapted with permission from ref. [86], Copyright 2018, Royal Society of Chemistry.

Following the large interest in Cuf‐amine based inks, Paquet et al. conducted an extensive study on the role that amine ligands play in determining the properties of the Cu films.^[86]^ Differing from previous studies, they isolated single crystals of their compounds and determined their structures via single‐crystal XRD, in order to present better chemical insight relating structure to decomposition profile. TGA curves of Cuf complexed with various primary and secondary amines suggested that the amines play an important role in lowering the activation energy of the formate decomposition, thereby lowering the decomposition temperature of these complexes (Figure 13). The ligands also act as capping agents during sintering, helping regulate particulate size. Complex decomposition temperature was also found to be dependent on the number of hydrogen bonds between the amines and formate ligands—intuitively the greater the number of hydrogen bonds, the higher the decomposition temperature. A comparison between the TGA curves of Cuf complexed with primary and secondary amines, as well as pyridines revealed the latter to have the lowest decomposition temperatures, whilst Cuf complex with primary amines have the highest.

Cuf complex inks have also been subject to sintering techniques other than thermal, such as laser, pulsed light and plasma sintering in order to obtain conductive films. For example, in 2013 Araki et al. laser sintered a Cuf‐1,2‐diethanolamine ink on glass,^[87]^ which resulted in a Cu film with *ρ=*5.60×10^−7^ Ω m. Lee et al. synthesised a Cuf ink complexed with hexylamine and AMP dispersed in isopropyl alcohol—subsequent laser sintering of the ink deposited on a PI surface resulted in a conductive film with *ρ=*1.70×10^−7^ Ω m.^[88]^ Drying the film before laser sintering was found to be essential to prevent the formation of oxides. Subsequently, Lee et al. published a morphological comparison of laser and thermal sintering of Cu films—while the laser sintered films were denser and thinner with a more uniform microstructure, the thermally sintered films exhibited better crystalline qualities which resulted in lower resistivities on PI substrates (*ρ=*1.70×10^−7^ Ω m and 1.92×10^−7^ Ω m for thermal and laser sintered films, respectively).^[89]^ Pre‐drying of the ink before sintering was found to be an essential step for both thermal and laser sintering. Interestingly, for both thermally sintered thinner and thicker films, films sintered at 150 °C (*ρ=*1.70×10^−7^ Ω m) were more conductive than those at higher temperatures, with the authors attributing this abnormal behaviour to the internal morphology and surface coverage of the annealed films.^[89]^ Min et al. synthesised a Cuf ink complexed with hexylamine and AMP which they sintered using a UV pulsed laser under N_2_ after drying the ink at 70 °C for 10 min, resulting in a Cu film on PI with *ρ=*1.74×10^−7^ Ω m.^[90]^ Farraj et al. sintered a Cuf‐AMP ink deposited on a PEN substrate via N_2_ plasma and were able to obtain highly conductive Cu films (*ρ*=7.30×10^−8^ Ω m) in 8 min (four times bulk Cu resistivity).^[91]^

While Cuf‐based MOD inks have garnered most of the interest in Cu MOD inks, the last decade has seen a few MOD inks synthesised using alternative Cu salts as starting reagents. Yang et al. synthesised copper glycolate and copper acetate‐cyclohexylamine inks.^[92]^ The printed films were first solidified in an oven at 60 °C for 2 h. Following this, the copper glycolate ink sintered at 290 °C for 1 h yielded a film with *ρ=*3.85×10^−7^ Ω m. The copper acetate‐cyclohexylamine ink sintered at 220 °C for 1 h yielded a conductive Cu film with *ρ=*7.50×10^−7^ Ω m on glass. Following this work, the same group reported the deposition of the copper acetate‐cyclohexylamine ink which formed conductive Cu onto an alkali treated PI substrate, which when sintered at 250 °C for 1 h yielded a film with *ρ=*2.20×10^−7^ Ω m.^[93]^

Deng et al. synthesised copper carboxylate inks, varying the length of the carboxylic acid chains.^[94]^ Carboxylic acids with shorter carbon chain lengths were shown to result in films with higher conductivity upon sintering, comparable to the results of Yang et al. above.^[92]^ Copper glycolate, lactate and oleate were used. While the longer chained copper oleate showed the poorest *ρ=*2.10×10^−4^ Ω m, copper glycolate and copper lactate resulted in far superior films with *ρ*=2.30×10^−7^ Ω m and 4.40×10^−7^ Ω m, respectively when sintered at 250 °C for 1 h on glass under N_2_. Draper et al. synthesised a copper nitrate‐hydroxide ink and sintered them with IPL.^[95]^ Sintering of the ink without additives resulted in decomposition of the copper nitrate complex to copper oxides and not the metallic element. To negate this, fructose and glucose were added as reducing agents. The film deposited with the copper nitrate‐hydroxide ink using fructose as the additive converted to metallic Cu and the film had a moderate *ρ=*1.25×10^−6^ Ω m.

**Figure 14:**
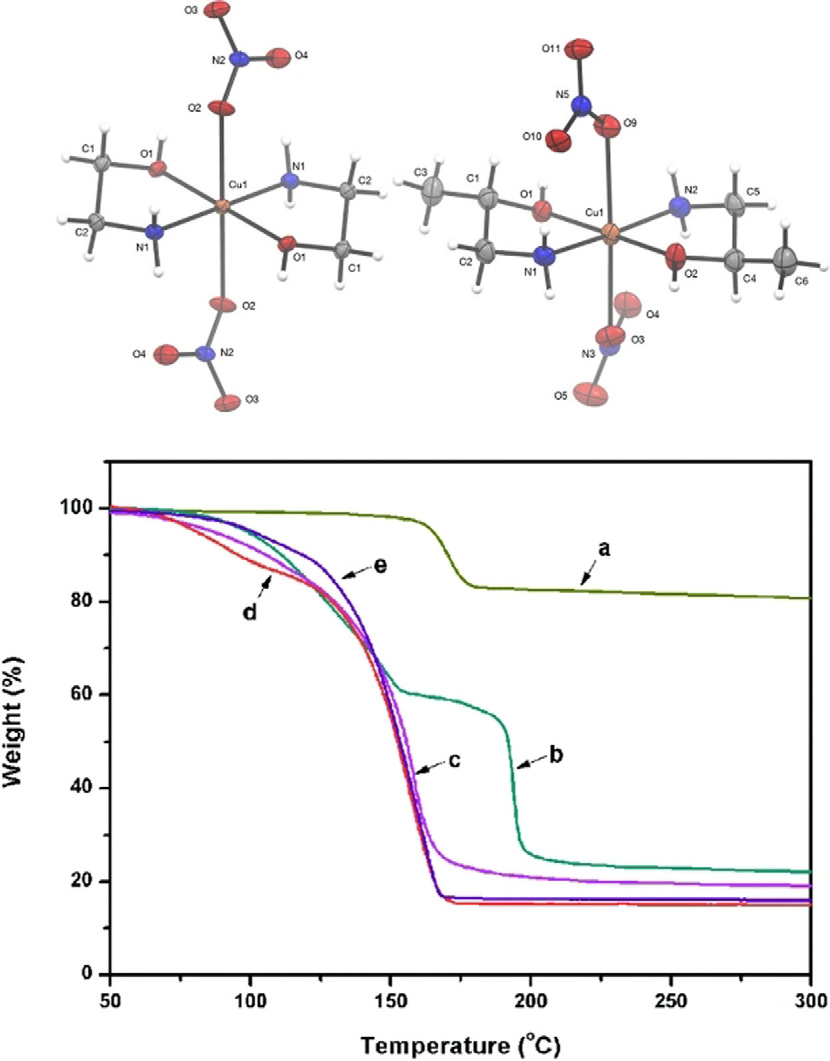
Top: molecular structure of MOD precursors: [Cu(EA)_2_(NO_3_)_2_] and [Cu(A2P)_2_(NO_3_)_2_]; bottom: TGAs of various Cuf‐diol complexes. (a) Cu(OH)_2_, (b) Cu(OH)_2_‐DMAPD, (c) Cu(OH)_2_‐DMAPD‐HCOOH, (d) Cu(OH)_2_‐DMAPD‐HCOOH (e) Cu(HCOO)_2_‐DMAPD. Adapted with permission from ref. [96], Copyright 2018 The Authors, and ref. [97], Copyright 2018 Royal Society of Chemistry.

More recently Knapp et al. synthesised bidentate diamine and alkanolamine stabilized copper nitrate complexes which were then dissolved in water to form inks.^[96]^ As with Paquet's study, the structures of the precursors were confirmed using single‐crystal XRD (Figure 14, top). Ethylenediamine, ethanolamine and amino‐2‐propanol were used as ligands and non‐thermal plasma sintering of these inks for 40 min resulted in films with *ρ*=1.50×10^−6^ Ω m, on glass. TGA data revealed significant changes to decomposition temperature depending on ligand choice in both Knapp et al.’s and Qi et al. contributions (Figure 14, bottom). The latter synthesised a copper hydroxide‐DMAPD ink.^[97]^ However, films deposited using these inks were not conductive. Formic acid had to be added as a catalyst to allow easy decomposition of the complex to metallic Cu. Using the formic acid catalyst films were deposited on glass and the lowest recorded *ρ*=1.39×10^−6^ Ω m was obtained, when sintered at 200 °C under N_2_.

Undoubtedly systems based on Cuf dominate the literature, with recent advances suggesting the use of different Cu salts as starting materials could warrant further investigation. Clear comparisons from Cu MOD research can be drawn with the Ag MOD literature, for example a broad array of sintering temperatures are used (with the usual inverse effect on the time taken) as well as reasonable shelf‐life data being presented.

#### 3.2.2 Copper NP inks

Research in the expanding field of Cu NP inks is vast. Not only are the inks varied in their method of synthesis (polyol,[^98^, ^99^, ^100^] reduction,[^101^, ^102^, ^103^] wire evaporation, seeded growth,^[104]^ etc.) but also various capping and dispersing agents are utilised. This Minireview addresses a selection of these innumerable inks that exist in literature, with the aim to cover the various capping agents that have been used so far and the effect the capping agent has on the sintering parameters and the final conductivity of the deposited Cu film. Although it is not the focus of this work, a number of articles have shown that CuO NP inks can be reduced to metallic Cu under harsh conditions.[^105^, ^106^, ^107^, ^108^]

Capping agents are a crucial factor in Cu NP inks as they help prevent oxidation of the NPs to copper oxides (Cu_2_O, CuO) during the sintering process. PVP is a commonly used capping agent which is either used by itself or in combination with cetyl trimethylammonium bromide (CTAB). Care must be taken when adding higher concentrations of capping agents in inks, such as PVP, as they are often non‐conductive and have elevated decomposition temperatures, with residual compound adversely affecting film performance. Thus, higher temperatures and longer times are required to ensure organic residues are removed upon sintering. Due to these competing effects, optimisation of the amount of capping agent added to the ink is crucial. As we have seen from Ag NP ink chemistry, carboxylic acids have attracted significant interest, which is likewise for its Cu counterpart, with the presence of these acids greatly helping prevent the formation of copper oxides. At the turn of the decade Woo et al.^[99]^ and Deng et al.^[109]^ made use of lactic acid as both a protecting and reducing agent, demonstrating experimentally how it reacts with copper oxides being formed during sintering, forming copper carboxylates which can be easily reduced in situ to metallic Cu. This paved the way for various other carboxylic acids to be used as additives in Cu NP inks.

Combining the advantages of PVP and carboxylic acids, Deng et al. were able to obtain a Cu film with a conductivity of 1.40×10^−7^ Ω m using an ink containing NPs capped with PVP and CTAB that were dispersed in lactic acid.^[110]^ This was obtained at a low sintering temperature of 200 °C under N_2_, however, a long sintering time of 30 min was required along with drying of films under vacuum for 1 h prior to annealing. Deng et al. also investigated other various short chain carboxylic acids as capping agents in Cu NP inks.^[103]^ Of these, they found lactic (used in their previous work) and glycolic acid rendered the best results. It was reported that the concentration of the carboxylic acid played a crucial role in controlling the size of the synthesised NPs, with smaller and more uniform NPs forming as acid concentration increased. Smaller NPs sinter at a lower temperature, whilst NP uniformity allows for denser packing, leading to the possibility of better conducting films at lower temperature. However, it must be noted that too high concentrations of acid can lead to large amounts of organic residue contaminating the deposited film, thereby hampering its conductivity, so optimisation is required. Using an ink with an optimised concentration of lactic acid capping agent, conductive films with *ρ*=9.10×10^−8^ Ω m were produced, after pre‐drying the ink for 1 h, followed by sintering at 200 °C for 1 h under a N_2_ atmosphere. The inverse relationship between resistivity and temperature that is common across all ink types was shown to be similar for these inks.

Kim et al. however, disagreed with the choice of lactic acid as it does not provide stability against oxidation for prolonged exposure,^[112]^ since no complete shell of capping agent fully covers the Cu NP. As a result, this study utilised formic acid for use as capping agent. This resulted in a copper‐formate core–shell formation as the surface oxides and hydroxides of the substrate reacted with the formic acid. This prevented oxidation of the NPs even after exposure to ambient conditions for extended periods of time. Formic acid formulations were also found to decompose at lower temperatures than their lactic acid counterparts (150 °C compared to 200 °C). However, the films deposited using this ink had higher resistivity compared to those that used lactic acid (*ρ*=1.35×10^−7^ Ω m) when heated at 250 °C for 1 h, likely owing to the formation of the core–shell structure. While the formation of a core–shell structure might provide better protection against oxidation, the higher organic content hampers coalescence, thereby reducing conductivity in the deposited films.

Oh et al. utilised oleic acid as the capping agent in their Cu NP ink,^[101]^ whilst comparing the benefits of using either thermal or photonic sintering using PI substrates. While thermal sintering yielded films with *ρ*=5.90×10^−8^ Ω m, photonic sintering yielded less conductive films with *ρ*=6.70×10^−8^ Ω m, however it must be noted that the photonic sintering was undertaken in the absence of any reducing/inert atmospheres. As the temperature of the substrate during photonic sintering did not exceed 110 °C, conductive films were also obtained on polyethersulfone (PES) and PET (*ρ*=1.91×10^−7^ Ω m and 5.12×10^−7^ Ω m, respectively). Also using oleic acid as the capping agent, Park et al. in 2016 synthesised a bimodal Cu NP ink,^[113]^ resulting in highly surface oxide free films. Employing laser welding, they were able to obtain films with *ρ*=4.60×10^−8^ Ω m on glass that were dried in air at RT prior to welding to allow solvent removal.

**Figure 15:**
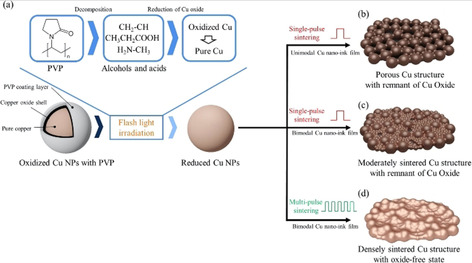
Decomposition pathway from Yu et al. Adapted with permission from ref. [111], Copyright 2017, IOP Science.

Yu et al. aimed to reduce the pores in—and hence improve conductivity of—their deposited films, by synthesising a bimodal NP ink (particles of diameter 40 nm and 100 nm) mixed together in PVP and ethylene glycol.^[111]^ It was hoped that the distribution of the smaller NPs in the spaces between the bigger NPs would result in more efficient packing of the NPs, leading to better conductivity. A ratio of 25:75 wt.% was found to be optimum for the smaller (40 nm) and larger (100 nm) particles. First the films were dried with near IR at 100 °C for 20 min and following flashlight sintering, films with *ρ*=5.68×10^−8^ Ω m, just over three times bulk Cu, were obtained on a PI substrate (Figure 15).

**Figure 16:**
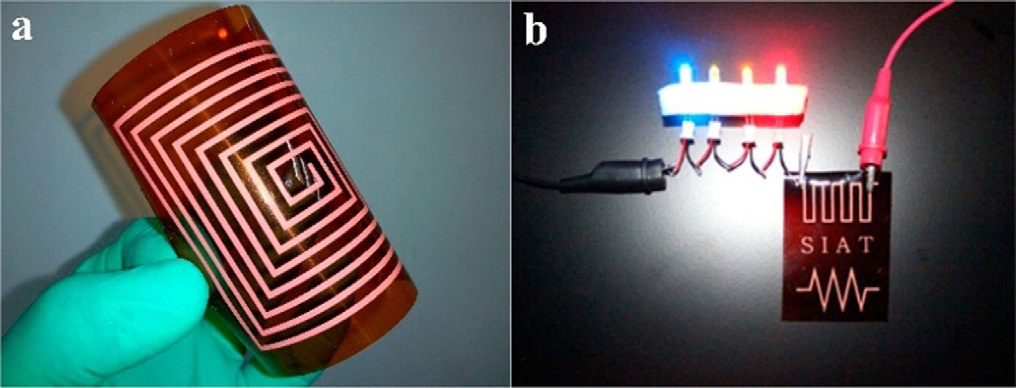
Conductive Cu circuit on flexible substrate using NP ink. Reproduced with permission from ref. [98], Copyright 2014, American Chemical Society.

Several other capping agents have also been used to synthesise Cu NP inks that have resulted in conductive films. Polyethylene glycol (PEG‐2000) was used as a protectant by Zhang et al.,^[98]^ along with l‐ascorbic acid as the reductant which aided in preventing oxidation of the NPs. Films deposited using this ink on PI were thermally sintered at 250 °C under a N_2_ environment for 30 min producing films with *ρ*=1.58×10^−7^ Ω m (Figure 16).

Another class of capping agent found in the Cu NP literature are alkanolamines. Hokita et al. synthesised small Cu NPs of diameter <10 nm that were stabilised with 1‐amino‐2‐propanol (AP), 3‐amino‐2‐propanol and ethylene glycol.^[114]^ The inks synthesised using the AP stabilised NPs showed the best results at low temperature. It was hypothesised that the secondary alcohol gets oxidised to a low BP ketone during the sintering process, which helps its removal whilst limiting contamination. The AP stabilised NP ink dried at 80 °C under N_2_ for 1 h followed by sintering at 150 °C for 15 min on PI resulted in films with *ρ*=3.00×10^−7^ Ω m. To lower the sintering time and enable sintering without the requirement of a reducing/inert atmospheres to make the process compatible with industrial roll‐to‐roll printing, working in the same group, Kanzaki et al. synthesised a NP ink with the Cu NPs stabilised by AP with added oxalic acid (1 wt.%) to aid oxidation free decomposition.^[115]^ Micron sized Cu flakes were also added to prevent cracking of the films. Sintering was carried out at 150 °C for 10 s in air. As clarified by X‐ray photoelectron spectroscopy (XPS), some oxidation was observed showing that sintering in air resulted in higher *ρ*=5.00×10^−7^ Ω m. Sintered films deposited from the same ink under N_2_ at 120 °C for 1 h resulted in a lower *ρ*=8.40×10^−8^ Ω m, ×4.5 times that of bulk Cu. The use of 2‐amino‐1‐butanol as the capping agent, another alkanolamine, by Sugiyama et al. from the same group resulted in a higher *ρ*=5.20 ×10^−8^ Ω m film when sintered at 150 °C for 30 min.^[116]^

Another sought after capping agent is graphene. Apart from preventing oxidation of the Cu NPs, being a conductive material graphene can also serve as conductive contact between the deposited NPs. However, the synthesis of graphene‐coated NPs is a multi‐step process, requiring high temperatures which has impeded its use in inks. Tseng et al. came up with a one‐step synthesis for graphene‐coated Cu NPs that were then used as inks for CVD onto various substrates.^[117]^ Films deposited on PI and silicon (Si) wafers were found to be conductive with *ρ*=1.70 ×10^−6^ Ω m and 1.40×10^−6^ Ω m, respectively. Inks developed using Cu NPs chelated with nitrilotriacetic acid (NTA) were also found to result in films with low resistivity.^[118]^ The inks were deposited on glass and sintered at varying temperatures for 30 min after optimising the amount of NTA to be added to the inks. Sintering at 200 °C resulted in films with *ρ*=1.60 ×10^−7^ Ω m, which further decreased to 1.00 ×10^−7^ Ω m when the sintering temperature was increased to 260 °C. Yokoyama et al. made use of trisodium citrate dihydrate as the capping agent in addition to varying amounts of ascorbic acid as the reducing agent.^[119]^ 60 wt.% Cu micron sized particles were also added to aid coalescence and films were obtained with *ρ*=8.20×10^−8^ Ω m when sintered at 300 °C for 1 h under argon (Ar) following drying under vacuum for 30 min. Li et al. also utilised trisodium citrate and CTAB as surfactants and were able to deposit conductive Cu films with *ρ*=7.20 ×10^−8^ Ω m on photopaper when heated under an Ar environment at 160 °C for 2 h.^[120]^

While Cu NP inks have received large interest, they still face issues with oxidation and the requirement to cap the NPs with various organic compounds increases the decomposition temperature of these inks. It seems that there is no ‘one size fits all’ capping agent, as the vast research would suggest.

### 3.3 Aluminium inks

To date, Al‐based metal inks are the least researched despite its low *ρ*=2.65×10^−8^ Ω m. Undoubtedly the biggest stumbling block when using Al to pattern metal, is its tendency to oxidise. MOD precursor species are typically very air‐sensitive, and as observed with MOD and NP inks alike: sintering steps require an inert/reducing atmosphere. Even so, Al's extremely low cost as the most abundant metallic element in the Earth's crust and its low work function make it an excellent candidate for conductive films and for electrodes for ohmic contact.

#### 3.3.1 Aluminium MOD inks

In the case of Al, there are more contributions to the MOD literature than NP, unlike as we have seen for Ag and Cu. This is likely owing to problems associated with oxide formation in Al NPs, as well as the suitability of high volatility Al organometallic complexes which initially prompted great interest in thin film deposition (of either the metal, or the oxide). Gladfelter and co‐workers pioneered the use of alanes (AlH_3_) as precursors for CVD, since their decomposition temperatures were considerably lower than Al−C bond containing molecules.^[121]^

Currently, the vast majority of Al MOD inks use Lewis base stabilised alane compounds (also seen as precursors to NP inks) as the Al source.^[122]^ Only over the past decade have these compounds been researched as precursors to the solution‐based deposition of metallic films, with the previous examples only featuring in patent literature, due to their industrially exciting applications.[^123^, ^124^]

Lee and co‐workers have made several remarkable contributions to the Al MOD ink field over the past decade. They first debuted their work in 2011,^[125]^ fabricating highly conductive Al films via a “warm solution‐stamping process ”, using *n*‐butyl ether stabilised alane (nBEA), [AlH_3_ **⋅**{O(*n*Bu)_2_}]. Films on both rigid and flexible substrates displayed outstanding electrical properties, with a best electrical resistivity achieved of only three times that of bulk Al (*ρ*=≈8.00×10^−8^ Ω m), at 150 °C for <1 min on glass. The ink was decomposed in the presence of evaporated titanium isopropoxide (TTIP): [Ti(O*i*Pr)_4_] (under an inert atmosphere). The use of TTIP became popular in subsequent work, often being described as a ‘catalyst’, although its recovery/regeneration is not clear. Experiments without a reducing ‘catalyst’ were also reported to thermally sinter at 165 °C. In 2012, Lee deposited Al films of ≈50 μm thickness onto numerous types of paper, using the same reactive sintering process.^[126]^ The best films were deposited onto inkjet printing paper, with lowest *R_s_* of ≤2 Ω sq^−1^. In 2013, Lee et al. employed a solution‐dipping process, again using nBEA, which allowed the preparation of larger area substrates.^[127]^ Lee dipped a pre‐heated (100 °C), pre‐treated (TTIP) PET substrate into the precursor solution at RT, producing a best film with *R*_s_=2.60 Ω sq^−1^, which only increased by a factor of ≈1.23 after 10 000 bending cycles.

**Figure 17:**
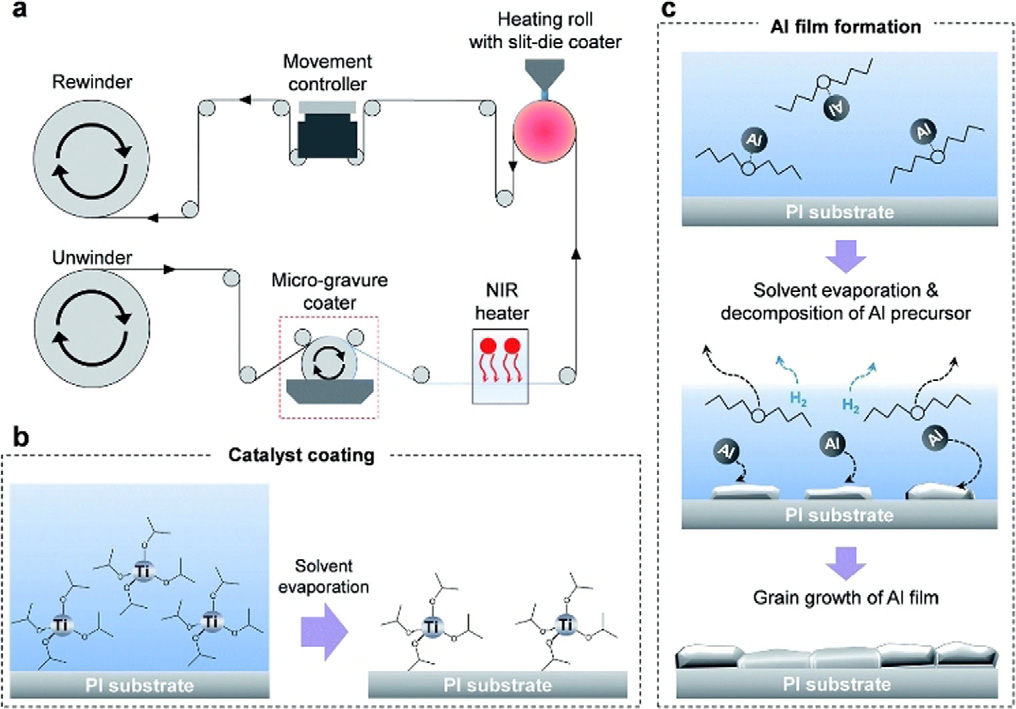
Lee's Al MODs: (a) Schematic of the roll‐to‐roll machine. Catalyst coating and Al precursor ink exposure are performed in one step. (b) Catalyst coating mechanism. (c) Al film growth mechanism, Al film is formed on the PI substrate in seconds. Reproduced with permission from ref. [129], Copyright 2018, Royal Society of Chemistry.

In 2015, Choi et al., working in the Lee group, were able to deposit Al metal on catalytically treated (TTIP) tissue paper with a high electrical conductivity (*R*_s_=≈0.700 Ω sq^−1^),^[128]^ which was used in high performance lithium‐sulfur batteries. Most recently in 2018, Lee's group (Jung et al.)^[129]^ took major steps in industrialising their work, by applying their alane precursor to roll‐to‐roll printing (under inert conditions) where they deposited Al onto PI using a diluted catalyst (100:1 volumetric ratio of dibutyl ether to TTIP). Films exhibited good durability and adhesion, producing a best metallic film that only was six times more resistant than bulk Al (*ρ*=1.70×10^−7^ Ω m) (Figure 17). Lee's solution stamping method of Al metal deposition using nBEA and TTIP has since been used for several applications other than for printed electronics, including for use in antimicrobial and electrostatic pollutant air filters,[^130^, ^131^] as well as wire grid polarizers.^[132]^

Fei et al. used a variation of Lee's process,^[133]^ depositing isopropyl ether stabilised alane (iPEA), [AlH_3_ **⋅**{O(*i*Pr)_2_}] in the presence of a titanium tetrachloride (TiCl_4_) reduction catalyst via a solution stamping‐process. The best metallic feature was sintered at 80 °C for 30 s to form a 50 nm thick cathode with *R_s_* 2.09 Ω sq^−1^ and a work function of 3.67 eV. They state that by swapping from *n*‐butyl ether to isopropyl ether, lower sintering temperatures were possible allowing them to deposit onto a larger range of substrates. They state that the more familiar diethyl ether alane analogue was not used as it is extremely flammable, even at temperatures as low as −5 °C.

nBEA forms highly thermally unstable solutions over time and as such Lee sought to remedy this by producing a more stable ink which was formulated using a more stable alane precursor, using an amine as the Lewis base.^[134]^ Amines are stronger Lewis bases than ethers due to the lower electronegativity of N compared to O and hence form stronger donor‐acceptor bonds to the alane moiety.

Trimethylamine alane (TMAA), [AlH_3_ **⋅**NMe_3_] was thus chosen. In addition to its higher stability, TMAA is solid at RT meaning it can be more easily isolated from its solvent during synthesis, allowing it to be stored solvent‐free which has several benefits. TMAA was dissolved in various solvents (diethyl ether, dibutyl ether, toluene) and the solution‐stamped films showed very similar resistivities, despite variable solubilities between solvents. All Al films deposited onto glass with the as‐prepared and 180 day stored precursor (−10 °C freezer) showed excellent electrical resistivities (*ρ*=5.80–7.00×10^−8^ Ω m), with a best of result of only twice as resistive as bulk metal. Shen et al. had previously used the amine stabilised alane; triethylamine alane (TEAA), [AlH_3_ **⋅**NEt_3_] as a precursor,^[135]^ however their solution processing required an expensive platinum catalysed, as well as high BP amines which helped to produce highly rough films with poorer conductivities.

Most recently in 2020, Douglas et al. took major steps in remedying the low Al wt.% loading found in MOD inks,^[136]^ by depositing two liquid amine stabilised alanes; dimethylethylamine alane (DMEAA), [AlH_3_ **⋅**NMe_2_Et] and TEAA neat on numerous substrates. Sintering temperatures as low as 100 °C were used, however the films with lowest resistivity were sintered at 120 °C. Depositions on paper were most adherent, whilst depositions on glass generally showed the best resistivity (4.25×10^−7^ Ω m, 120 °C, 30 s).

In summary it is evident that this emerging field produces results comparable to Ag and Cu, with recent reports showing that catalytic additives can now be removed. However, currently this area is limited by its use of extremely reactive precursors and the need for an inert atmosphere.

#### 3.3.2 Aluminium NP inks

Presently, most commercially available metal NP inks are Ag or Cu based. The problem of NP oxidation which is seen in Cu, is amplified when using Al as the source metal, as it suffers drastically from the highly thermodynamically stable surface oxide layer forming. When used in the bulk, the native oxide of Al (≈3–5 nm) is negligible, however in NPs, the surface‐to‐volume ratio of oxide to metal limits electrical performance. Thus, it is essential to use surface coatings to prevent surface oxidation, which has also been seen in Cu NP chemistry, where materials such as carbon,^[137]^ organic surfactants,^[138]^ metal salts^[112]^ and secondary metals^[139]^ have been used. A bonus of using organic surfactants as capping agents is that they aid in preventing aggregation and precipitation. A similar capping approach curbing Al reactivity has been applied to Al NP‐based fuels and rocket propellants.[^140^, ^141^]

Lee at al. are one of the only groups to research Al NPs for use in conductive inks.^[122]^ In 2018, Al NPs were synthesised using an adapted version of the Haber and Buhro method,^[142]^ using DMEAA as the Al source and TTIP as the decomposition catalyst. Oleic acid was used to cap the NPs, with particle size dependent on the injection time. Surface capping was confirmed via XPS and also showed the absence of an oxide layer. The prepared NPs were stored in a low humidity desiccator for 2 days prior to XPS analysis, meaning that the oxidation stability study of NPs in this work was limited to 2 days in air. Conductive films were deposited using a 30 wt.% solution in toluene via spin coating, with films sintered in a reducing atmosphere (10 % H_2_ with 90 % Ar) at temperatures between 200 °C–600 °C. All synthetic procedures were conducted in an Ar filled glove box to prevent oxidation. The best deposits were at 600 °C with lowest *ρ=*4.12×10^−7^ Ω m, approximately ten times greater than bulk Al, as this temperature allowed for the greater coalescence of particles. Whilst these processing temperatures are high, the work function of the exemplar film was calculated to be 4.22 eV, proving that Al NPs are more efficient than noble metals such as Ag and Cu for use in electrodes.

## 4 Summary and Outlook

As we have seen, the deposition of inks via inkjet printing, spin coating and other similar methods can lead to fast, low temperature conversions onto a broad range of substrates: from glass, to plastic and cloth, yielding highly conductive features, finding use in a multitude of electronic devices. The main focus of this Minireview has been to consider the synthetic routes towards the array of inks that have populated the literature in the past decade. Until recently the MOD precursor field was limited by lower metal wt.% loading despite better shelf life and tuneability via chemical structural design, which has potential to extend the number of compounds used. NP inks dominate industry and literature alike and whilst shelf life and stability can be problematic higher wt.% loading acts to reduce environmental impact and contamination.

In the printing of conductive Ag, Walker et al. achieved the lowest conductivity in 2012 and since that time improvements have been made to shelf life and processing temperatures and times. Altering sintering type has brought temperatures down to ambient and conversion times from hours to seconds. Fast expansion in the area of NP capping agents has yielded conductive features, albeit at relatively high temperatures. Recent advances in morphological control through varying particle size has scope for the future. This Minireview has highlighted isolated MOD precursors as an emergent area, currently most advanced for Cu, where crystalline ‘made‐to‐measure’ compounds can be stored indefinitely and then used in inks. The conversion of these crystalline compounds to metal can be tracked using XRD, potentially offering untold insight to deposition mechanisms. This avenue has gone some way to mitigating Cu's high susceptibility to oxidation in air, with MOD precursors designed to circumvent issues. The Al MOD ink field shows the beginnings of real‐world application with routes toward industrial scale up plausible, if the problems of air‐sensitivity can be resolved.

Methods of capping to make NPs less prone to oxidation have been reported to much success in the field of Cu inks, and lessons should be learnt and applied the area of Al NP inks—this area is ready for investigation. It would seem that features can be printed on a broad array of substrates, with the field moving away from glass and plastics and onto paper and fabrics in the last decade. In many areas the issue of adhesion still remains, and since it varies considerably from substrate to substrate, is not a problem easily remedied. Critical challenges that limit the performance include the inverse relationship between sintering temperature/time and resistivity; so new strategies may need to be considered to go beyond the current limits. From Ag to Cu and Al, MOD and NP formulations have been shown to deposit highly conductive features, but many are still not inkjet printed—this represents a challenge for the future: application of inks to this ideal technique.

## Conflict of interest

The authors declare no conflict of interest.

## Biographical Information

*Samuel Douglas is in the final year of his PhD under the supervision of Dr Caroline Knapp. He studied his undergraduate MSci Chemistry degree at UCL where he gained the Nyholm award for most distinguished work in Inorganic Chemistry. As a postgraduate student he was awarded an Inorganic/Materials sectional award (Gillespie Scholarship), as well as the prize for best progressing graduate (Tufnell Scholarship). Sam's research is currently centred on the synthesis of reactive Al compounds as precursors to conductive metal coatings*.

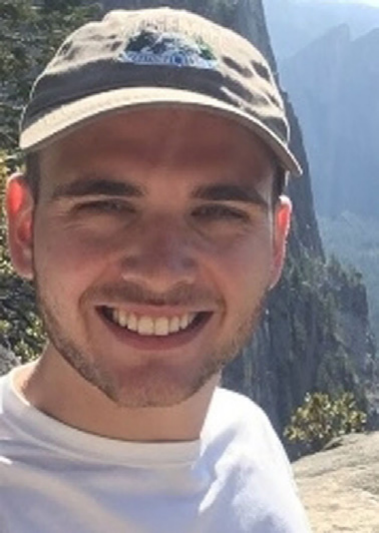

## Biographical Information

*Shreya Mrig is a 2nd year PhD student at UCL under the supervision of Dr Caroline Knapp. She received her MSci in Chemistry from UCL in 2018, for which she was awarded a place on the Dean's List. Her PhD research is focused on the design of precursors such that their decomposition can be fine‐tuned. She specialises in Cu and Al complexes that can be used to inkjet print highly conductive tracks for use in flexible electronics*.

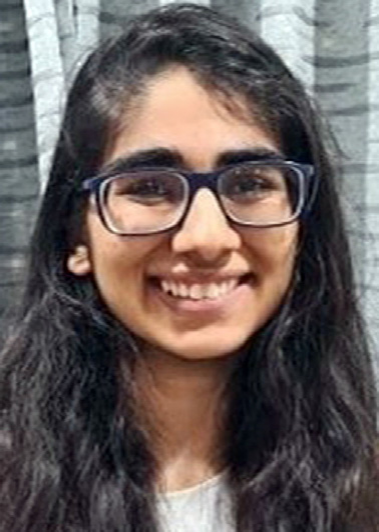

## Biographical Information

*Dr Caroline Knapp is Lecturer in Molecular Inorganic Chemistry at UCL. Prior to this she held a Ramsay Memorial Fellowship alongside a visiting position at the Luxembourg Institute of Science and Technology, funded by an Intermobility grant. She gained her MSci and PhD from UCL under the supervision of Prof Claire J. Carmalt. She worked as a postdoctoral researcher with Prof Phil P. Power FRS (UC Davis) and Dr Joachim Steinke (Imperial College). Her laboratory now carries out research designing and isolating precursors for the printing of metals*.

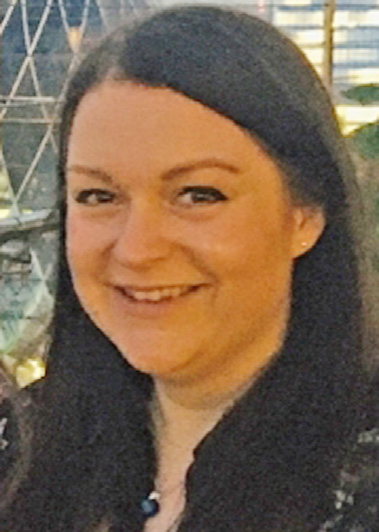

## Supporting information

As a service to our authors and readers, this journal provides supporting information supplied by the authors. Such materials are peer reviewed and may be re‐organized for online delivery, but are not copy‐edited or typeset. Technical support issues arising from supporting information (other than missing files) should be addressed to the authors.

Supplementary
