# Supplementary material for: MODs vs. NPs: Vying for the Future of Printed Electronics
Source: Chemistry. 2021 Mar 1;27(31):8062–81. doi: 10.1002/chem.202004860 (PMC8247916; doi:10.1002/chem.202004860)
Supplement: Supplementary file 1 — Supplementary [file CHEM-27-8062-s001.pdf]

Chemistry–A European Journal

Supporting Information

MODs vs. NPs: Vying for the Future of Printed Electronics

Samuel P. Douglas+, Shreya Mrig+, and Caroline E. Knapp*[a]

The tables below represent the data for every ink listed in the Minireview. For ease of comparison, all conductivity values have been converted to volume resistivity in units of Ω m (*note:* Ω m = (S $m^{-1})^{-1}$). Sheet resistance values have not been converted as information is not always given as to the volume of the films. Inks appear in order of their mention and all acronyms are defined in the main text of the Minireview. Detailed descriptions of substrate pre-treatments are not listed here.

Table S1. Summary of Ag MOD inks

Ink composition	Substrate/s	Deposition technique	Sintering Conditions	Resistivity/Resistance	Reference
Diamminesilver(I) + formic acid	PET, PI, glass, cellulose-based materials.	Direct-writing, inkjet printing and airbrush spraying	Thermal: 90 °C, 15 min	$\rho = 1.60 \times 10^{-8}$ Ω m	Walker[16]
Diamminesilver(I) + formic acid	PI	Drop-coating	Direct laser patterning of ink	$\rho = 2.10 \times 10^{-8}$ Ω m	Liu[18]
Diamminesilver(I) + formic acid	Polyacrlyonitrile<br>Polypropylene<br>PET<br>Basalt<br>Cotton	Inkjet printing	Thermal: 90 °C, 8 printing and sintering cycles	$R_s = 0.169$ Ω sq^{-1}<br>0.177 Ω sq^{-1}<br>0.155 Ω sq^{-1}<br>0.235 Ω sq^{-1}<br>0.389 Ω sq^{-1}	Stempien[19]
Diamminesilver(I) + formic acid<br>Diamminesilver(I) + formic acid + 0.5 wt.% PVP	Poly(ethylene dioxythiophene):poly(styrene sulfonate) (PEDOT:PSS) buffer layer	Flexography printing	Thermal: 90 °C, 15 min	$R_s = 0.700$ Ω sq^{-1}<br>7.20 Ω sq^{-1}	Iannaccone[20]
Diamminesilver(I) + formic acid	Paper	Inkjet printing	Ar/H_2 plasma sintering at RT, 200 printing and sintering cycles totalling 6 min 40 s	$\rho = 2.60 \times 10^{-8}$ Ω m.	Knapp[21]
Diamminesilver(I) + formic acid	PI	Inkjet printing	Thermal: 90 °C, 15 min, followed by plasma irradiation for 4 min<br>Thermal only<br>Plasma only	$\rho = 6.00 \times 10^{-8}$ Ω m<br>1.20×10^{-7} Ω m<br>1.00×10^{-6} Ω m	Wang[22]
Silver ammonia + diethanolamine	PET	Inkjet printing	Thermal: 75 °C, 20 min	$\rho = 6.00 \times 10^{-8}$ Ω m	Chen[23]

Silver-ethylamine-ethanolamine-formate complex	Glass<br>PEN	Inkjet printing	Thermal: 150 °C, 30 min	$\rho = 4.10 \times 10^{-8}\ \Omega$ m<br>$4.70 \times 10^{-8}\ \Omega$ m	Vaseem[24]
Silver-ethylamine-ethanolamine-formate complex	PI	Inkjet printing	Thermal: 80 °C, 5 min, 8 printing and sintering cycles. , followed by 80 °C for 30 min	$\rho = 5.95 \times 10^{-8}\ \Omega$ m	Vaseem[25]
Silver-ethanolamine-formate	Glass	Spin-coating	Microwave-plasma sintering for 1.5 min	$\rho = 7.14 \times 10^{-8}\ \Omega$ m	Vaseem[26]
Silver-ethylamine-ammonia	Glass<br>PET	Spin-coating<br>Inkjet printing	Thermal: 60 °C, 1 day<br>Thermal: 75 °C, 1 h	$\rho = 9.32 \times 10^{-7}\ \Omega$ m<br>$3.65 \times 10^{-7}\ \Omega$ m	Bhat[27]
Silver-isopropanolamine	PI	Mask printing	Thermal: 110 °C, 2 h	$\rho = 1.21 \times 10^{-7}\ \Omega$ m	Mou[28]
Silver neodecanoate + ethyl cellulose	PI<br>PI<br>Glass	Screen printing<br><br>Aerosol jet printed	Photonic sintering after drying 75 °C, 15 min<br>Thermal, 230 °C, 10 min after drying 140 °C, 15 min<br>Thermal: 220 °C, 15 min, 10 min after drying 140 °C, 15 min	$\rho = 2.00 \times 10^{-7}\ \Omega$ m<br>$8.80 \times 10^{-8}\ \Omega$ m<br>$2.80 \times 10^{-8}\ \Omega$ m	Kell[29]
Silver neodecanoate + cellulose	Glass	Direct-writing	Thermal: 115 °C, 1 h	$\rho = 9.00 \times 10^{-8}\ \Omega$ m	Shen[30]
Silver-diethanolamine-acetate	Glass, PET	Spin-coating	Thermal: 100 °C, 10 min, followed by 180 °C, 30 min, 2 layers	$\rho = 6.67 \times 10^{-8}\ \Omega$ m	Xie[31]
Silver citrate + 1,2-diaminopropane	PET<br>Glass	Inkjet printing<br>Drop-coating	Thermal: 150 °C, 50 min<br>Thermal: 230 °C, 50 min	$\rho = 1.70 \times 10^{-7}\ \Omega$ m<br>$3.10 \times 10^{-8}\ \Omega$ m	Nie[32]
Silver citrate + ethylenediamine	PI	Drop-coating	Thermal: 200 °C, 1 h	$\rho = 3.90 \times 10^{-7}\ \Omega$ m	Yang[33]

Silver-amine-oxalate	PI	Drop-coating	Thermal: 160 °C, 1 h	$\rho = 8.19 \times 10^{-8}$ Ω m	Yang[34]
Silver trifluoroacetate + DMAc + butanone	SIS	Direct pen writing	Multiple cycles of in-situ chemical reduction, 5 min per cycle	Line resistance: 80.0 Ω m^{-1}	Hu[35]
[(hfac)(1,5-COD)Ag]	Glass	Inkjet printing	Thermal: 120 °C, ~30 min (15 × ~2 min printing and sintering cycles)	$\rho = 4.10 \times 10^{-8}$ Ω m	Black[36]

Table S2. Summary of Ag NP inks

Ink composition	Substrate/s	Deposition technique	Sintering (Pre-sintering) Conditions	Resistivity/Resistance	Reference
Short chain carboxylic acids (C6/C10) capped Ag NPs – C10i ink	PI, Glass, Paper	Aerosol jet printing	Thermal: 250 °C, 91 h 30 min	$\rho = 1.83 \times 10^{-8}$ Ω m	Ankireddy[42]
Short chain carboxylic acid (C10) capped Ag NPs	PI	Aerosol jet printing	Thermal: 240 °C, 1 h	$\rho = 3.38 \times 10^{-8}$ Ω m	Ankireddy[43]
Oleic acid capped Ag NPs	PI<br>PET	Drop-coating	Photonic: 2.0 kV, 2 ms (dried at RT for 10 min)<br>2.0 kV for 1.5 ms (dried at RT for 10 min)	$\rho = 5.10 \times 10^{-8}$ Ω m<br>8.00×10^{-8} Ω m	Jo[44]
Humic acid capped Ag NPs	Photo-paper	Inkjet printing	Thermal: 180 °C, 1 h, 40 printed layers	$\rho = 1.35 \times 10^{-6}$ Ω m	Hao[45]
Dodecylamine capped Ag NPs	PI	Aerosol jet printing	Thermal: 300 °C, 1 h	$\rho = \sim 1.59 \times 10^{-7}$ Ω m*	Shankar[46]
Alkylamine capped Ag NPs	Photoactivated polymer	Blade-coating	Chemical: reduction with irradiated polymer surface<br>No post-processing<br>Post processing at < 80 °C	$\rho = \sim 1.00 \times 10^{-6}$ Ω m<br>1.00×10^{-7} Ω m	Yamada[47]

Ag NPs capped with octylamine	Photo-paper	Direct pen writing	Thermal: 100 °C, 1 h	$\rho = 3.06 \times 10^{-7}$ Ω m	Ghosale[49]
PAA capped Ag NPs	Photo-paper	Inkjet printing	Thermal: 180 °C, 15 min, (air drying for 1 day)	$\rho = 3.70 \times 10^{-8}$ Ω m	Shen[51]
PAA-alkanolamine capped Ag NPs	PI	Screen printing	Thermal: 250 °C, 1 h	$\rho = 5.60 \times 10^{-8}$ Ω m	Shao[52]
PAA capped Ag NPs	PET	Screen printing	Thermal: 120 °C, 1 h	$\rho = 1.52 \times 10^{-7}$ Ω m	Huang[53]
PAA capped Ag NPs	Platinum coated Si wafers	3D printing	Thermal: 250 °C, 1 h	$\rho = < 10^{-6}$ Ω m	Lee[55]
CHBP capped Ag NPs	Photo-paper	Inkjet printing	Thermal: 180 °C, 1 h 20 min, 30 printed layers	$\rho = 10.83 \times 10^{-8}$ Ω m	Hao[56]
PVP capped Ag NPs	PET	Screen printing	Thermal: 160 °C, 1 h 15 min	$\rho = 3.83 \times 10^{-8}$ Ω m	Ding[57]
PVP capped Ag NPs	Photo-paper	Inkjet printing	Thermal: 300 °C, 30 min	$\rho = 5.60 \times 10^{-8}$ Ω m	Fernandes[58]
Sodium citrate capped Ag NPs	Glass	Blade-coating	Thermal: 180 °C, 20 min	$\rho = 1.56 \times 10^{-7}$ Ω m	Yin[59]
In situ fabrication of Ag NP inks	Cotton fabric	Dip coating	Thermal: 200 °C, 30 min	Rs: 5.20 Ω sq^{-1}	Kardarian[62]

Sodium citrate capped Ag NPs	Cotton fabric	Screen printing	Thermal: 60 °C, 30 min, in presence of HCl catalyst	$\rho = 2.00 \times 10^{-5}\ \Omega$ m	Wang[63]
PAA Na stabilized Ag NPs	PET	Inkjet printing	Self-sintering at RT using NaCl as destabilizer<br>Chemical: HCl vapor as destabilizer, 10 s	$\rho = 1.60 \times 10^{-7}\ \Omega$ m<br>$3.84 \times 10^{-8}\ \Omega$ m	Grouchko[54]
Bimodal Ag NPs ink	Photo-paper	Inkjet printing	Air drying at RT, 17 printing and drying cycles	$\rho = 3.66 \times 10^{-8}\ \Omega$ m	Liu[65]
Polyvinyl bromide capped Ag NPs	Paper	Direct- drawing	Thermal: 110 °C, 5 h	$\rho = 6.70 \times 10^{-8}\ \Omega$ m	Murtaza[66]

[a] *Values have been extrapolated from graphs in the research paper.

Table S3. Summary of Cu MOD inks

Ink composition	Substrate/s	Deposition technique	Sintering (Pre-sintering) Conditions	Resistivity/Resistance	Reference
Cuf + *α*-terpinol + dispersion agent	PI	Screen printing	Laser, under N_2	$\rho = 1.86 \times 10^{-7}\ \Omega$ m	Joo[72]
Cuf + *α*-terpinol + dispersion agent	PI	Screen printing	Thermal: 275 °C, 1 h, under N_2<br>Laser, under N_2	$\rho = 1.30 \times 10^{-7}\ \Omega$ m<br>$\rho = 1.41 \times 10^{-7}\ \Omega$ m	Joo[73]
Cuf+ PVP + ethylene glycol + 2-methoxyethanol	PI, PET	Rollerball pen	IPL	$\rho = 4.62 \times 10^{-8}\ \Omega$ m	Wang[74]
Cuf in distilled water	Glass	Reactive transfer printing	Thermal: 200 °C, 30 min; under N_2	$\rho = 3.29 \times 10^{-8}\ \Omega$ m	Rosen[75]

Cuf + SWCNT + PVP	PI	Screen printing	IPL (dried at 70 °C for 5 min)	Resistance: 9.26 Ω	Rosen[76]
Cuf + *n*-octylamine	Glass	Screen printing	Thermal: 140 °C, 1 h, under N_2 (dried at RT for 30 min)	$\rho = 2.00 \times 10^{-7}$ Ω m	Yabuki[77]
Cuf + 20:80 blend of dibutylamine and *n*-octylamine	Glass	Screen printing	Thermal: 140 °C, 30 min, under N_2	$\rho = 5.00 \times 10^{-8}$ Ω m	Yabuki[78]
Cuf + AMP + octylamine + hexanoic acid	Glass	Drop-coating	Thermal: 200 °C, 30 min, under N_2<br>350 °C, 30 min, under N_2	$\rho = 2.34 \times 10^{-7}$ Ω m<br>9.46×10^{-8} Ω m	Shin[79]
Cuf + DEAPD	Glass	Screen printing	Thermal: 180 °C, 5 min	$\rho = 3.00 \times 10^{-6}$ Ω m	Yabuki[80]
Cuf + AMP (hydrated)<br>Cuf + AMP (dehydrated)	Glass, PET, PEN	Screen printing	Thermal: 190 °C, under N_2	$\rho = 1.05 \times 10^{-7}$ Ω m<br>4.50×10^{-7} Ω m	Farraj[15]
Cuf + 1:1 butylamine:octylamine	Glass<br>PI<br>PET<br>PEN	Drop-coating	Thermal: 200 °C, 40 min<br>180 °C, 20 min<br>160 °C, 20 min<br>160 °C, 20 min	$\rho = 4.28 \times 10^{-8}$ Ω m<br>9.69×10^{-8} Ω m<br>2.29×10^{-7} Ω m<br>2.14×10^{-7} Ω m	Xu[14]
Cuf + monoisopropanolamine	Glass		Thermal: 140 °C, 5 min, under N_2	$\rho = 2.00 \times 10^{-7}$ Ω m	Huang[81]
Cuf + 2 wt% water + 95:5 2-ethylhexylamine:AMP	PI	Drop-coating	Thermal: 250 °C, 30 min, under N_2	$\rho = 5.20 \times 10^{-8}$ Ω m	Xu[82]
Cuf + 1,2-diaminepropane	PI	Drop-coating, spin-coating, direct pen writing	Thermal: 180 °C, 1 min	$\rho = 1.80 \times 10^{-7}$ Ω m	Dong[83]

Cuf + pentylamine	Glass	Screen printing	Thermal: 110 °C, 30 min, under N_2	$\rho = 5.70 \times 10^{-8}$ Ω m	Yabuki[84]
Cuf + 60:40 3-butylpyridine:2-ethyl-1-hexylamine	PEN	Doctor-blade-coating	Thermal: 170 °C, < 5 min, under N_2	$\rho = 6.50 \times 10^{-8}$ Ω m	Paquet[85]
Cuf + 1,2-diethanolamine	Glass	Mask printing	Laser	$\rho = 5.60 \times 10^{-7}$ Ω m	Araki[87]
Cuf + hexylamine + AMP	PI	Spin-coating	Laser (dried at 70 °C for 10 min)	$\rho = 1.70 \times 10^{-7}$ Ω m	Lee[88]
Cuf + hexylamine + AMP	PI	Spin-coating	Laser (dried at 70 °C for 10 min)<br>Thermal, 150 °C, 1 h furnace cycle to RT	$\rho = 1.92 \times 10^{-7}$ Ω m<br>$\rho = 1.70 \times 10^{-7}$ Ω m	Lee[89]
Cuf + hexylamine + AMP	PI	Spin-coating	Laser under N_2 (dried at 70 °C for 10 min)	1.74×10^{-7} Ω m	Min[90]
Cuf-AMP	PEN	Screen printing	N_2 plasma, 8 min (dried 100 °C for 10 min)	7.30×10^{-8} Ω m	Farraj[91]
Copper glycolate + cyclohexylamine<br>Copper acetate + cyclohexylamine	Glass<br>Glass	Drop-coating	Thermal: 290 °C, 1 h, under N_2 (dried in oven at 60 °C for 2 h)<br>220 °C, 1 h (dried in oven at 60 °C for 2 h)	$\rho = 3.85 \times 10^{-7}$ Ω m<br>7.50×10^{-7} Ω m	Yang[92]
Copper acetate +cyclohexylamine	PI	Drop-coating	Thermal: 250 °C, 1 h, under N_2	$\rho = 2.20 \times 10^{-7}$ Ω m	Yang[93]
Copper oleate<br>Copper glycolate<br>Copper lactate	Glass	Solution-drop-coating	Thermal: 250 °C, 1 h, under N_2	$\rho = 2.10 \times 10^{-4}$ Ω m<br>2.30×10^{-7} Ω m<br>4.40×10^{-7} Ω m	Deng[94]

Copper nitrate hydroxide + fructose	Ryonet mesh grid	Screen printing	IPL	$\rho = 1.25 \times 10^{-6}\ \Omega$ m	Draper[95]
Copper nitrate + ethylenediamine/ethanolamine/amino-2-propanol	Glass	Inkjet printing	Ar/H_2 plasma, 40 min	$\rho = 1.50 \times 10^{-6}\ \Omega$ m	Knapp[96]
Copper hydroxide DMAPD + formic acid	Glass	Blade coating	Thermal: 200 °C, 30 min, under N_2	$\rho = 1.39 \times 10^{-6}\ \Omega$ m	Qi[97]

Table S4. Summary of Cu NP inks

Ink composition	Substrate/s	Deposition technique	Sintering (Pre-sintering) Conditions	Resistivity/Resistance	Reference
Cu NPs capped with PVP & CTAB, lactic acid as protectant	Glass	Drop-coating	Thermal: 200 °C, 30 min, under N_2 (dried in vacuum for 1 h)	$\rho = 1.40 \times 10^{-7}\ \Omega$ m	Deng[110]
Cu NPs capped with lactic acid	Glass	Pastes deposited on slides	Thermal: 200 °C, 1 h, under N_2 (dried under vacuum for 1 h)	$\rho = 9.10 \times 10^{-8}\ \Omega$ m	Deng[103]
Cu NPs capped with formic acid	Glass	Spin-coating	Thermal: 250 °C, 1 h, under N_2	$\rho = 1.35 \times 10^{-7}\ \Omega$ m	Kim[112]
Cu NPs capped with oleic acid	PI<br>PI<br>PES<br>PET	Airbrush printing	Thermal<br>Photonic: 2.5 kV, 1.5 ms<br>Photonic: 2.0 kV, 1.5 ms<br>Photonic: 2.3 kV, 1.5 ms	$\rho = 5.90 \times 10^{-8}\ \Omega$ m<br>$6.70 \times 10^{-8}\ \Omega$ m<br>$1.91 \times 10^{-7}\ \Omega$ m<br>$5.12 \times 10^{-7}\ \Omega$ m	Oh[101]
Bimodal Cu NP ink capped with oleic acid	Glass	Spin-coating	Laser (dried in air at RT)	$\rho = 4.60 \times 10^{-8}\ \Omega$ m	Park[113]
Bimodal Cu NPs capped with PVP	PI	Screen printing	Flashlight sintering (dried using near IR at 100 °C for 20 min)	$\rho = 5.68 \times 10^{-8}\ \Omega$ m	Yu[111]

PEG-2000 protected Cu NPs	PI	Screen printing	Thermal: 250 °C, 30 min, under N_2	$\rho = 1.58 \times 10^{-7}\ \Omega$ m	Zhang[98]
Cu NPs stabilized with AP	PI	Bar-coating	Thermal: 150 °C, 15 min, under N_2 (dried at 80 °C for 1 h under N_2)	$\rho = 3.00 \times 10^{-7}\ \Omega$ m	Hokita[114]
Cu NPs stabilized with AP + oxalic acid + Cu flakes	PI	Doctor-blade-coating	Thermal: 120 °C, 1 h, under N_2	$\rho = 8.40 \times 10^{-8}\ \Omega$ m	Kanzaki[115]
2-amino-1-butanol protected Cu NPs	PI	Bar-coating	Thermal: 150 °C, 30 min, under N_2	$\rho = 5.20 \times 10^{-7}\ \Omega$ m	Sugiyama[116]
Graphene coated NPs	PI<br>Si wafer	CVD	CVD maintained at 230 °C for 2 h	$\rho = 1.70 \times 10^{-6}\ \Omega$ m<br>$1.40 \times 10^{-6}\ \Omega$ m	Tseng[117]
Cu NPS chelated with NTA	Glass	Screen printing	Thermal: 260 °C, 30 min, under N_2	$\rho = 1.00 \times 10^{-7}\ \Omega$ m	Kamikoriyama[118]
Cu NPs capped with trisodium citrate dihydrate	Glass	Doctor-blade-coating	Thermal: 300 °C, 1 h, under Ar (dried for 30 min under vacuum)	$\rho = 8.20 \times 10^{-8}\ \Omega$ m	Yokoyama[119]
Cu NPs + trisodium citrate + CTAB	Photo-paper	Direct pen writing	Thermal: 160 °C, 2 h, under Ar	$\rho = 7.20 \times 10^{-8}\ \Omega$ m	Li[120]

Table S5. Summary of Al MOD and NP inks

Ink composition	Substrate/s	Deposition Technique	Sintering Conditions	Resistivity/Resistance	Reference
nBEA + TTIP	Glass, PET	Solution-stamping in Ar glovebox	Chemical and thermal: TTIP substrate, 150 °C, <1 min, under Ar	$\rho =\sim 8.00 \times 10^{-8}\ \Omega$ m*	Lee[125]

nBEA	Paper	Solution-stamping in Ar glovebox	Chemical and thermal: TTIP treated substrate, 110 °C, 1 min, under Ar	$R_s \leq 2\ \Omega\ sq^{-1}$	Lee[126]
nBEA	PET	Solution-dipping	Chemical: TTIP substrate, solution-dipped at RT for 20 min, dried at RT, under Ar	$R_s = \sim 2.60\ \Omega\ sq^{-1}$	Lee[127]
nBEA	Tissue paper	Solution-dipping	Chemical: TTIP substrate solution-dipped for 2 h, dried at RT, under Ar	$R_s = \sim 0.700\ \Omega\ sq^{-1}$	Choi[128]
nBEA	PI	Slit-die-coating (roll-to-roll printing)	Chemical and thermal: TTIP treated substrate 150 °C, web speed of 3 m min^{-1}, under Ar	$\rho = 1.70 \times 10^{-7}\ \Omega$ m	Jung[129]
iPEA	PET	Solution-stamping	Chemical and thermal: $TiCl_4$ treated substrate, 80 °C, 30 s, under inert conditions	$R_s = 2.09\ \Omega\ sq^{-1}$	Fei[133]
TMAA	Glass	Solution-stamping	Chemical and thermal: TTIP treated substrate, 130 °C, 1 min, under Ar	$\rho = 5.80–7.00 \times 10^{-8}\ \Omega$ m	Lee[134]
TEAA	Substrate patterned with platinum nanocrystals	Solution-dipping	Thermal: 105–120 °C, 30 min–2 h, under N_2	$\rho = 2.80–3.50 \times 10^{-8}\ \Omega$ m	Shen[135]
TEAA	Glass	Drop-coating	Thermal: 120 °C, 30 s, under N_2	$\rho = 4.25 \times 10^{-7}\ \Omega$ m	Douglas[136]
Oleic acid capped Al NPs	Glass	Spin-coating	Thermal: 600 °C, under Ar/H_2 environment	$\rho = 4.12 \times 10^{-7}\ \Omega$ m	Lee[122]

[a] *Values have been extrapolated from graphs in the paper.
